# Calcium and Calcineurin-NFAT Signaling Regulate Granulocyte-Monocyte Progenitor Cell Cycle via Flt3-L

**DOI:** 10.1002/stem.1813

**Published:** 2014-11-26

**Authors:** Jan Fric, Clarice XF Lim, Alexandra Mertes, Bernett TK Lee, Elena Viganò, Jinmiao Chen, Francesca Zolezzi, Michael Poidinger, Anis Larbi, Herbert Strobl, Teresa Zelante, Paola Ricciardi-Castagnoli

**Affiliations:** a Singapore Immunology Network (SIgN), Agency for Science, Technology and Research (A*STAR)Singapore; b Institute of Pathophysiology and Immunology, Medical University GrazAustria; c Department of Medicine I, Institute for Cancer Research, Medical University of ViennaAustria; d Department of Biological Sciences, National University of SingaporeSingapore

**Keywords:** Flt3 signaling, Cyclosporine A, Hematopoiesis, Myeloid differentiation, Tacrolimus

## Abstract

Maintenance of myeloid progenitor cells is controlled by complex regulatory mechanisms and is orchestrated by multiple different transcription factors. Here, we report that the activation of the transcription factor nuclear factor of activated T cells (NFAT) by calcium-sensing protein calcineurin inhibits the proliferation of myeloid granulocyte–monocyte progenitors (GMPs). Myeloid progenitor subtypes exhibit variable sensitivity to induced Ca^2+^ entry and consequently display differential engagement of the calcineurin-NFAT pathway. This study shows that inhibition of the calcineurin-NFAT pathway enhances the proliferation of GMPs both in vitro and in vivo and demonstrates that calcineurin-NFAT signaling in GMPs is initiated by Flt3-L. Inhibition of the calcineurin-NFAT pathway modified expression of the cell cycle regulation genes *Cdk4, Cdk6*, and *Cdkn1a* (*p21*), thus enabling rapid cell cycle progression specifically in GMPs. NFAT inhibitor drugs are extensively used in the clinic to restrict the pathological activation of lymphoid cells, and our data reveal for the first time that these therapies also exert potent effects on maintenance of the myeloid cell compartment through specific regulation of GMP proliferation. Stem Cells *2014;32:3232–3244*

## Introduction

Regulation of myeloid hematopoiesis plays a key role in the maintenance of innate immune responses. The nuclear factor of activated T cells (NFAT) family of transcription factors has been recently been identified as an important player in the renewal of various myeloid cell subsets 1,2. The NFAT family has five members, of which the activation cascade of NFAT1-4 is driven by increased levels of intracellular Ca^2+^. Ca^2+^ is sensed by calmodulin, which activates calcineurin-mediated dephosphorylation of NFAT resulting in translocation of NFAT into the nucleus 3–5. Apart from its crucial and well-described role in embryogenesis 6,7 and T cells 3, the calcineurin-NFAT pathway controls several innate immune functions of dendritic cells (DCs), macrophages, mast cells, megakaryocytes, and osteoclasts 2,8. NFAT signaling also regulates apoptosis of terminally differentiated DCs 9, further promoting maintenance of the steady-state. In contrast, during infection, the calcineurin-NFAT pathway is required for effective neutrophil responses to *Candida* 10 and effective macrophages responses to *Leishmania* 11.

As well as being involved in myeloid cell functions, NFAT now appears to be important for myeloid compartment development 1 and megakaryopoiesis 12. Although myeloid cells are present in mice lacking calcineurin-NFAT signaling 13, NFAT deficiency leads to progressive abnormalities including extramedullary hematopoiesis in the spleen and reduced numbers of hematopoietic stem cells (HSCs) in bone marrow (BM) 14. In humans, there is parallel evidence for involvement of NFAT in the differentiation of immunomobilized CD34^+^ HSCs 15. However, in each case, the mechanisms underlying these effects of NFAT are unknown. Myeloid hematopoiesis proceeds from HSCs, through multipotent progenitors (MPPs), common myeloid progenitors (CMPs), and finally to GMPs that give rise to fully committed myeloid cells 16. Within the myeloid lineage, NFAT negatively regulates the differentiation of megakaryocytes 12,17 and induces development of osteoclasts 18. Genes regulating the cell cycle have been identified as NFAT targets in T cells, 19 stem cells 20, and in embryonic development and lineage specification 21, but not in the myeloid compartment. Indeed, the main networks regulating proliferation and differentiation in hematopoietic progenitors have also been identified 22, but a role for NFAT in the maintenance of myeloid progenitor cells has not previously been reported.

The hematopoietic process is controlled by growth factors and cytokines, including SCF, IL-3, and IL-6 23, and specifically, in the case of myeloid cells by G-CSF, M-CSF, GM-CSF 24,25, and Flt3-L 16,26,27. Interestingly, SCF, IL-3, IL-6, and GM-CSF signaling all increase the levels of intracellular Ca^2+^ in HSCs 28–30. Furthermore, IL-3 and GM-CSF signaling are associated with phospholipase Cγ (PLCγ_2_), the main driver of increases in intracellular Ca^2+^ levels 30, and M-CSF 25 and G-CSF 31 phosphorylate PLCγ_2_ in BM progenitors, while also being critical determinants of cell lineage commitment. Whether induction of Ca^2+^ release in any of these instances results in NFAT activation is unknown, as are the potential downstream effects on cell function. It has been shown that Flt3 ligation activates PLCγ_2_ 26,32, but the association with Ca^2+^ release, calcineurin and NFAT translocation are currently unknown. The possible link between Flt3/Flt3-L signaling and NFAT induction is particularly intriguing since Flt3-L is a key growth factor for hematopoietic progenitors and also initiates the main signaling pathway responsible for in vivo steady-state differentiation of DCs 33–37. Flt3 is expressed on MPPs, CMPs, and GMPs 16,36,37, while also sustaining progenitor expansion 38,39, and promoting the growth of colony-forming units (CFU) 40. Flt3-L is a key cytokine responsible for both development of myeloid cells 33,34,41 and promotion of inflammatory immune responses 42. Furthermore, expression of Flt3-L has been reported on GMPs 36,37 and Flt3 signaling is known to directly impact on GMP development 43; however, the mechanism underlying this process remains poorly defined.

Here, we report that NFAT is both present and functional within myeloid progenitors, and directly inhibits the proliferation of GMPs. In addition, we reveal that NFAT mobilization can be triggered by Flt3-L signaling specifically in GMPs, thus providing compelling evidence of a role for NFAT in myeloid hematopoiesis, which has direct implications for the therapeutic inhibition of NFAT in the clinic.

## Materials and Methods

### Animals

C57BL/6, C57BL/6-ppp3r1^tm1stl^/J-(Cnb1^flox^), Mx1-cre, and CD45.1 mice were from The Jackson Laboratory and were maintained under specific pathogen-free conditions. Cyclosporine A (CsA) (4 mg) or FK506 (0.4 mg) were injected intraperitoneally. C57BL/6(B6)-CD45.1/CD45.2 heterozygote recipients were lethally irradiated with 12Gy in two doses separated by 4 hours and reconstituted with five million BM cells consisting of a mixture of 50% Cnb1^flox/flox^Mx1-cre and 50% age- and sex-matched CD45.1. Cells from mice were analyzed 8 weeks after reconstitution. To induce Cnb1-knock out (KO) in Cnb1^flox/flox^Mx1-cre mice, animals were treated with five intraperitoneal injections of 250 μg of polyI:C (Invivogen) every other day for 10 days. All mice were bred in the Biological Resource Centre (A*STAR, Singapore) and handled according to institute guidelines under the approval of the Institutional Animal Care and Use Committee.

### Flow Cytometry and Sorting of BM Progenitor Populations

Lineage marker positive BM cells were depleted using biotinylated antibodies (CD45R, CD3e, Gr-1, CD19, NK1.1, TER-119, CD127, and CD11b) and streptavidin-microbeads using a cell separator (AutoMACS-Miltenyi, http://www.miltenyibiotec.com). In some experiments, progenitors were further enriched with cKIT beads. Progenitors were labeled with anti-mouse antibodies: PB-c-Kit (CD117), FITC-CD16/32, PE-Flt3 (CD135), AF647-Sca-1 (Ly6A/E), AF700-CD34. and PE-Cy7 streptavidin. The lineage-depleted cells were sorted into progenitor subsets: HSCs (Lin^−^, c-Kit^+^, Sca-1^+^, CD34^+^,Flt3^−^), MPPs (Lin^−^, c-Kit^+^, Sca-1^+^, CD34^+^, Flt3^+^), CMPs (Lin^−^,Sca-1^−^,c-Kit^+^, CD34^+^, CD16/CD32^int^). and GMPs (Lin^−^, Sca-1^−^, c-Kit^+^, CD34^+^, CD16/CD32^hi^), using an Aria II cell sorter.

### PLCγ Phosphorylation Analysis by Flow Cytometry

Lineage-negative cells were cultured for 4 hours and then stimulated for 2 or 10 minutes with rmFlt3-L (1μg/ml). Progenitor subsets were labeled with antibodies, cells were fixed and incubated with anti-pPLCγ_1_-FITC and total PLCγ_1_-PE and analyzed using the FlowCellect PLCγ_1_ Activation Dual Detection Kit (Merck Millipore, Billerica, MA, www.emdmillipore.com) according to manufacturer's instructions. Alternatively, cells were fixed and incubated with anti-pPLCγ_2_-FITC (BD Phosphoflow, BD Bioscience, Mississauga ON, www.bdbiosciences.com), according to the kit instructions, using Phosphoflow Perm Buffer III.

### Intracellular Ca^2+^ Mobilization Assay

Sorted GMP, CMP, and lin^−^Sca1^+^cKIT^+^ BM cells (LSKs), pooled HSCs and MPPs) progenitors (1.5 × 10^4^ cells per well) were plated in a black 384 wells-plate (Perkin Elmer, Waltham, MA, www.perkinelmer.com) and rested at 37°C with 5% CO_2_ for 3 hours. The cells were then incubated for 45 minutes in dark condition with 20 μl of Hanks' balanced salt solution (HBSS) containing HEPES 20 mM, probenecid 2.5 mM and Fluo4-NW (Life Technologies, Invitrogen, Carlsbad, CA, www.lifetechnologies.com). Fluorescence were measured with spectrophotometer Victor^4^ (Perkin Elmer) (excitation, 485 nm; emission, 535 nm) every second for 180 seconds after injection of the stimuli (diluted in HBSS): Flt3L (4 μg/ml), ionomycin (500 ng/ml), thapsigargin (2 μM). The intracellular calcium chelator BAPTA (10–20 μM, Invitrogen) was added to the culture 1 hour before the incubation with Fluo4-AM. The entire experiments were performed at 37°. The values (F) were normalized by the first point (F0) after the injection of the stimuli and the percentage (F/F0*100) is shown. Alternatively, lineage-depleted BM cells were loaded with Indo-1 AM (2 mM; Life Technologies, Molecular Probes, Carlsbad, CA, www.lifetechnologies.com) by incubation at 37°C for 20 minutes, and Ca^2+^ release monitored by measuring the ratio of signal from INDO-1 (Violet) BP 525/50 versus INDO-1 (Blue) BP 450/50 on a LSR II for 1 minute followed by 4 minutes after adding the trigger Flt3-L (2 μg/ml), ionomycin (500 ng/ml), thapsigargin (2 μM). Cells were kept in at a temperature of 37°C until the measurement. Kinetics was analyzed using FlowJo software.

### Luciferase Assay

Lineage-depleted cKIT^+^ cells or the HSC line were transduced using Cignal Lenti NFAT Reporter (luc) Kit (Quiagen, SabBiosciences, Venlo, Netherlands, www.sabiosciences.com), using Transcriptional Regulatory Element Sequence GGA GGA AAA ACT GTT TCA TAC AGA AGG CGT according to protocol. Luciferase activity was detected 4 hours after trigger using ONE-Glo Luciferase Assay System (Promega, Madison, WI, www.promega.com).

### Cell Culture

BM cells were cultured in IMDM (containing 200 ng/ml mrFlt3-L (Stem cells), 10% heat-inactivated FCS (Life Technologies), streptomycin 100 mg/ml, and penicillin 100 U/ml) at 3 × 10^6^cells per milliliter. For culture of sorted progenitors, stem cell factor (SCF; 50 ng/ml), IL-6 (20 ng/ml), and IL-3 (10 ng/ml; all R&D Systems, Minneapolis, MN, http://www.rndsystems.com) were added (referred to as HSC medium). Two micrograms per milliliter CsA or 0.2 µg/ml FK506 (Cell Signaling Technology, Danvers, MA, www.cellsignal.com) were added to the cell culture for 30 minutes preceding the addition of Flt3-L/GM-CSF and were maintained throughout the culture.

### Isolation and Culture of Primary Human CD34^+^ Cells

CD34^+^ umbilical cord blood cells (CB) were obtained from full-term healthy deliveries after informed consent and purified as described previously 44,45. Cells were cultured in 24-well plates (2 × 10^4^ cells per well) in serum-free X-vivo 15 medium (BioWhittaker, Lonza, Walkersville, MA, www.lonza.com) supplemented with 100 ng/ml Flt3-L in presence or absence of CsA (2μg/ml). Approval was obtained from the Medical University of Vienna Institutional Review Board.

### Immunofluorescence Labeling

GMPs were fixed after sorting or cultured for 24 hours in HSC medium followed by stimulation with ionomycin (500 ng/ml) for 15 minutes. Cells were fixed with paraformaldehyde (2%) before permeabilization in 0.5% Saponin, and blocking for 1 hours with 3% bovine serum albumin. Cells were incubated for 1 hours (37°C) with anti-NFAT2 antibody (10 µg/ml) (Thermo Scientific, Waltham, MA, www.thermoscientific.com) followed by secondary antibody (AF633 goat anti-mouse IgG, Life Technologies, Molecular Probes) at 1 µg/ml and DAPI at 2 µg/ml. Cellular localization of NFAT2 was visualized using an Olympus FV1000 confocal microscope.

### CFU Assay

Mouse BM cells were cultured at 4 × 10^4^ cells per milliliter in MethoCult M3534 (StemCell Technologies, Vancouver, BC, www.stemcell.com). MethoCult (500 µl per well) were plated in a 12-well suspension culture plate (Greiner, Sigma Aldrich, St. Loius, MO, www.sigmaaldrich.com) and incubated at 37°C in 5% CO_2_. Colonies were counted under the light microscope after 5–7 days.

### Cell Proliferation Analysis

For in vitro bromodeoxyuridine (BrdU) assays, cells were pulsed with 1 mM BrdU for 1 hours. For in vivo BrdU assays, mice were injected with CsA (4 mg/mouse), FK506 (0.4 mg/mouse), or vehicle. At the 48 hours time-point, mice were killed and BM analyzed. BrdU (1.5 mg/mouse) was injected 1 or 18 hours before analysis. BM was lineage-depleted and progenitors populations labeled and gated as described in the sorting strategy above. Cells were fixed and labeled using a BrdU flow cytometry kit (BD Biosciences). Proliferation was also assessed by CFSE dilution (Life Technologies, Molecular Probes), cells were labeled with 2 μM CFSE following the manufacturer's protocol.

### Quantitative Real-Time PCR

Total cellular RNA was extracted by Trizol (Invitrogen) phase separation followed by purification using RNeasy Mini/Micro kit (Qiagen), or by using the *Arcturus PicoPure* RNA Isolation Kit. Reverse transcription was carried out using high-capacity cDNA Reverse Transcription Kit with RNase Inhibitor (Applied Biosystems), or with SuperScript III First Strand Synthesis System for RT-polymerase chain reaction (PCR) (Invitrogen). Real-time PCR was carried out with primers listed in the Supporting Information using GoTaq qPCR Master Mix (Promega).

### Microarray Hybridization and Analysis

Total RNA was extracted using a double extraction protocol. ssDNA was prepared, fragmented, and labeled according to the Affymetrix protocol. Fragmented ssDNAs were hybridized to the standard arrays for 17 hours at 45°C; the arrays were then washed and stained using the fluidics station and then scanned using GeneChip Scanner 3000. The gene expression data were then filtered for only probes where the associated gene had a valid NCBI Entrez Gene ID to restrict data to well annotated genes. Gene ontology terms were used to identify genes involved in regulation of cell cycle and transcriptional regulation of differentiation and hematopoiesis. These genes were then tested using a series of two-way analysis of variance (ANOVA) to identify genes that differed in their expression levels due to time or treatment. Processing of the data used Accelrys Pipeline Pilot with visualizations in TIBCO Spotfire. All microarray data files are available for free download at the Gene Expression Omnibus (GEO accession number: GSE47208, http://www.ncbi.nlm.nih.gov/geo. Detailed procedure is described in Supporting Information Methods.

### Statistical Analysis

Unless specified differently in the legend, all values are shown as means ±SEM. Student's *t*-test was used to identify significant differences between groups. For all tests, the 0.05 confidence level was considered statistically significant. In figures, *denotes *p* < .05, **denotes *p* < .01, and ***denotes *p* < .001 in an unpaired Student's *t*-test.

## Results

### Calcineurin-NFAT Inhibitors Cyclosporin A and FK506 Selectively Increase Proliferation and Numbers of GMPs In Vivo

**Figure 1:**
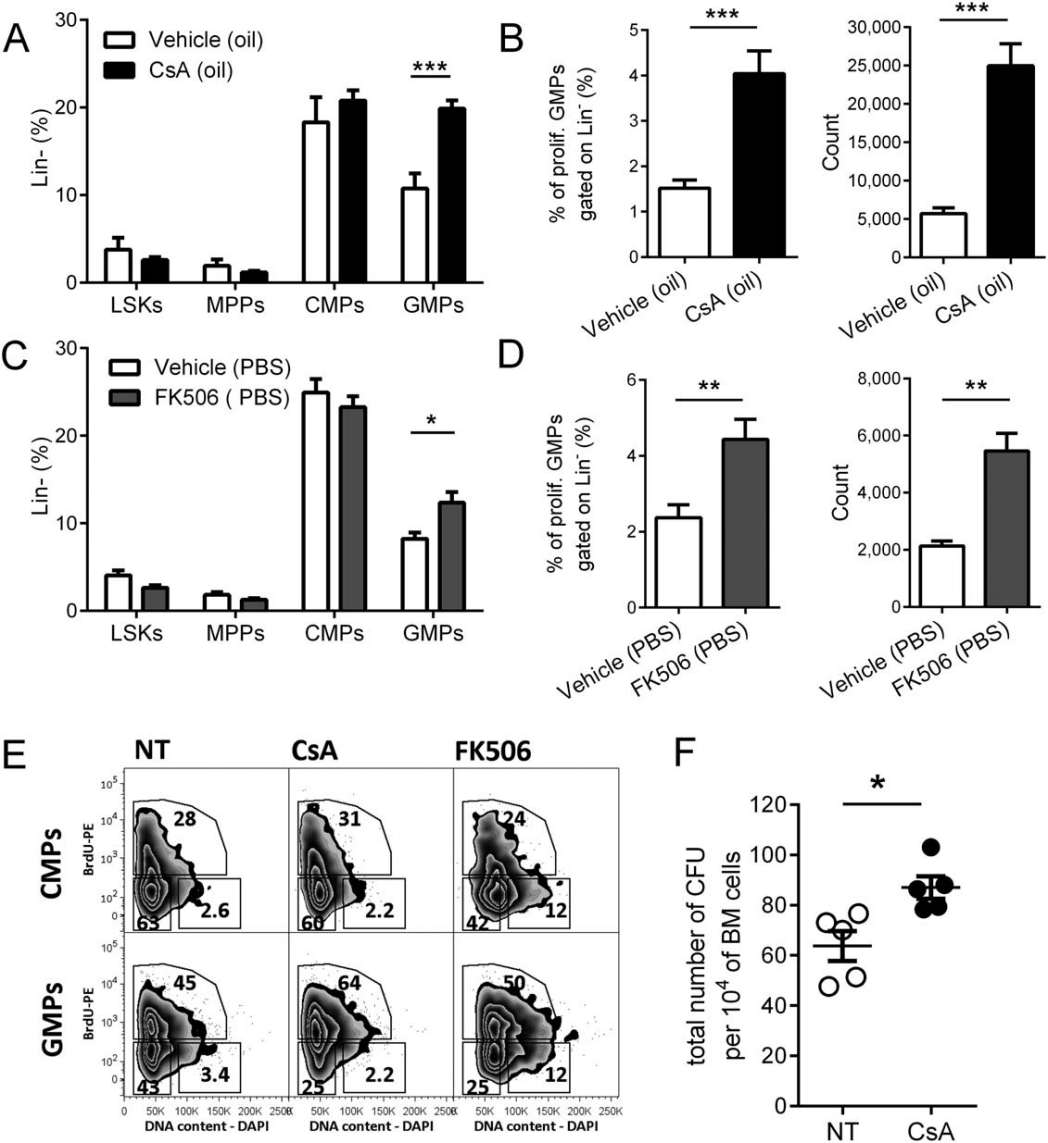
Calcineurin-nuclear factor of activated T cells (NFAT) inhibition results in increased granulocyte–monocyte progenitor (GMP) proliferation in vivo. (A–D): Quantification of the percentage of hematopoietic progenitors in bone marrow (BM) upon in vivo treatment with Cyclosporine A (CsA) (A, B) and FK506 (C, D). Progenitor populations identified as LSKs (lin^−^, cKit^+^, Sca-1^+^), multipotent progenitors (MPPs) (lin^−^, cKit^+^, Sca-1^+^, CD34^+^, FLT3^+^), common myeloid progenitors (CMPs) (lin^−^, cKit^+^, Sca-1^−^, CD34^+^, CD16/32^int^), and GMPs (lin^−^, cKit^+^, Sca-1^−^, CD34^+^, CD16/32^high^). Percentage of progenitors among lineage negative cells from mice treated with CsA (A) and FK506 (C). (B, D): Detailed analysis of percentage and cell numbers of GMPs from mice treated with CsA (B) and FK506 (D). Mean ± SE from two independent experiments out of five is shown. At least three mice per group (vehicle, CsA, or FK506) were analyzed in each experiment, *, *p* < .05; **, *p* < .01; and ***, *p* < .001 in an unpaired Student's *t*-test. (E): Cell cycle analysis in CMPs (lin^−^, cKit^+^, Sca-1^−^, CD34^+^, CD16/32^int^) and GMPs (lin^−^, cKit^+^, Sca-1^−^, CD34^+^, CD16/32^high^) isolated from mice injected with CsA, FK506, or vehicle and bromodeoxyuridine (BrdU). Representative plot of cell cycle analysis gated for CMPs and GMPs is shown. Dividing cells in S phase (BrdU^+^) and M phase (DNA content - DAPI^+^) were further analyzed. Representative experiment is shown, five independent experiments were performed with 3–5 mice per experimental group. (F): Number of myeloid progenitors in BM. BM cells were plated in methylcellulose and resulting colonies counted 5–7 days later. Total number of colony-forming units (CFU) (myeloid progenitors) per 10^4^ BM cells is plotted. One representative experiment of three is shown. Mean ± SE is plotted, *n* = 5; *, *p* < .05 in an unpaired Student's *t*-test. Abbreviations: BrdU, bromodeoxyuridine; CFU, colony-forming units; CMP, common myeloid progenitors; CsA, Cyclosporine A; DAPI, 4′,6-diamidino-2-phenylindole; GMP, granulocyte–monocyte progenitor; LSK, lin^−^Sca1^+^cKIT^+^ bone marrow cells; MPP, multipotent progenitors; NT, non-treated; PBS, phosphate-buffered saline; PE, phycoerythrin.

We have previously observed that calcineurin inhibitors enhance myelopoiesis 1. To identify which hematopoietic progenitors are regulated by calcineurin-NFAT signaling, mice were treated with the calcineurin-NFAT inhibitor drugs CsA or Tacrolimus (FK506). After 48 hours of CsA treatment, we detected no change in the percentage of HSCs, MPPs and CMPs among lineage-negative BM cells, whereas percentages of GMPs were significantly increased (Fig. 1A, 1E). The proliferation rate of GMPs was then assessed by BrdU incorporation and DNA content analysis (Fig. 1B). The percentage and total number of proliferating BrdU-positive GMPs (Fig. 1B) was significantly increased in CsA-treated mice. Significant effects of calcineurin-NFAT inhibition on GMP proliferation were also observed with the alternative drug inhibitor FK506 (Fig. 1C–1E). Supporting Information Figure 1A shows the gating strategy used. Increased cell cycle progression upon in vivo administration of both CsA and FK506 was specifically observed in GMPs, whereas CMPs were not affected (Fig. 1A, 1C, 1E). Treatment with calcineurin-NFAT inhibitors did not significantly change the total numbers of BM cells or splenocytes (Supporting Information Fig. 2A, 2B), while numbers of LSKs, MPPs, CMPs and GMPs in BM show similar trend as percentage obtained after both inhibitors treatment (Supporting Information Fig. 2C). Accordingly, when BM cells were cultured in methylcellulose medium containing SCF, IL-3, and IL-6, BM from mice treated with CsA gave rise to significantly higher numbers of granulocytic and monocytic CFU (CFU-GM) than did the BM of untreated animals (Fig. 1F).

These data indicate that GMPs but not LSKs, MPPs, or CMPs exhibit increased proliferation after inhibition of the calcineurin-NFAT pathway in vivo.

### Calcineurin (*Cnb1*) Deficiency in HSCs Increases the Frequency of GMPs In Vivo

**Figure 2:**
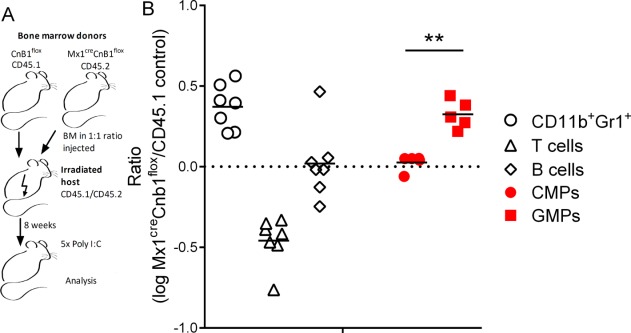
Granulocyte–monocyte progenitors (GMPs) deficient in calcineurin specifically expand in vivo over and above control counterparts. (A–B): Bone marrow (BM) chimeric mice were generated using CD45.1 control and Cnb1^flox/flox^Mx1-cre (CD45.2) BM cells mixed in 1:1 ratio as BM graft. After engraftment (8 weeks) the knock out was induced by PolyI:C injection. (A): Experimental schema showing BM from Cnb1^flox/flox^Mx1-cre (CD45.2) and control (CD45.1) donors mixed in 1:1 ratio and injected into irradiated hosts (heterozygotes CD45.1/CD45.2). (B): Different cell subpopulations (CD11b^+^Gr1^+^—myeloid cells, CD4^+^ T cells, and CD19^+^ B cells) in black were gated and changes in the donor ratio depicted as logarithm of Cnb1^flox/flox^Mx1-cre frequency divided by CD45.1 control frequency. Log of ratio for common myeloid progenitors (CMPs) and GMPs is shown in red indicating specific-change in GMPs. Original injected ratio 1:1 is 0 (black line). Ratio of each subpopulation from individual mice is shown. Representative of two independent experiments, with at least five mice in each is shown. Supporting Information Fig. 3A depicts gating strategy. Mann-Whitney test (**, *p* < .01) was used to compare changes in ratios of GMPs and CMPs. Abbreviations: BM, bone marrow; CMP, common myeloid progenitors; GMP, granulocyte–monocyte progenitor.

To confirm the effects of calcineurin-NFAT signaling on GMP proliferation in vivo, we next generated mice that harbor a conditional knockout of the regulatory subunit of calcineurin (*Cnb1, Ppp3r1*) 46 in all hematopoietic cells. Cnb1^flox/flox^ animals 47 were crossed with Mx1-cre mice expressing inducible cre-recombinase 48. Cnb1 knockout in HSCs in Cnb1^flox/flox^Mx1-cre mice was then induced using polyI:C injections. Irradiated recipient mice (heterozygote CD45.1/CD45.2) underwent hematopoietic reconstitution with a 1:1 mix of BM cells from Cnb1^flox/flox^Mx1-cre (CD45.2) and control (CD45.1) donors (Fig. 2A; Supporting Information Fig. 3A, 3B). After full engraftment, Cnb1 knockout was induced with polyI:C treatment, as depicted in Figure 2A. The origin and frequency of different progenitors and differentiated cells from Cnb1 knockout and control donors were measured using CD45.1 and CD45.2 expression. The ratio of Cnb1 knockout versus control origin within different cell populations was assessed by flow cytometry. Supporting Information Figure 3A illustrates the gating strategy used. Figure 2B reveals the ratio between Cnb1^flox/flox^Mx1-cre (CD45.2) and control donor (CD45.1) cells in each gated subset (CD11b^+^Gr1^+^ myeloid/granulocytic cells, CD4^+^ T cells, CD19^+^ B cells, CMPs, and GMPs). We observed significant increases in the frequency of GMPs originating from Cnb1^flox/flox^Mx1-cre BM. In contrast, CMPs and B cells remained at the original injected ratio of 1:1 cells derived from the Cnb1-knockout and control BM. Reconstituted mice also exhibited increased numbers of CD11b^+^Gr1^+^ and CD11c^+^MHCII^+^ cells in calcineurin-NFAT-impaired animals, while in contrast the majority of T cells were recruited from the control donor. Similar changes were also reflected in total cell numbers counts (Supporting Information Fig. 1D). Ratio of engrafted donor cells was 1:1 (black line, Fig. 2B). Poly I:C-induced knockout of Cnb1 was confirmed in sorted Cnb1^flox/flox^ Mx1-cre (CD45.2) DCs and CD11b^+^Gr1^+^ myeloid cells by comparing expression levels with control (CD45.1) cells (Supporting Information Fig. 3B). Furthermore, we validated knockout of Cnb1 in magnetic bead-enriched Ly6G^+^, CD11c^+^, and CD4^+^ cells obtained from Cnb1^flox/flox^Mx1-cre mice compared with cells isolated from littermate controls (Cnb1^flox/flox^Mx1-wt) (Supporting Information Fig. 3C).

These results suggest that impaired calcineurin signaling confers GMPs with a proliferation advantage that impacts downstream myeloid differentiation.

### CsA Promotes the Proliferation of Flt3-L Stimulated Human CD34^+^ Umbilical Cord Blood Cells In Vitro

**Figure 3:**
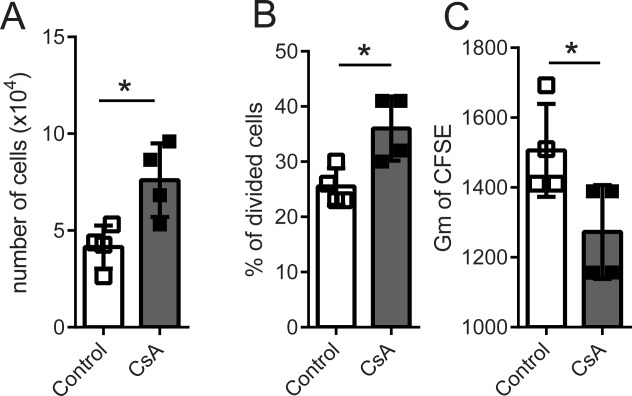
Cyclosporine A (CsA) promotes the proliferation of Flt3-L dependent human hematopoietic progenitors cells. CD34^+^ cells were loaded with CFSE dye and cultured for 3 days with Flt3-L in presence or absence of CsA (2 μg/ml). Total cell numbers (A), percentage of divided cells (B), and mean of CFSE (C) are shown. Data from four donors are shown, mean ± SE and individual value for each donor are plotted, *, *p* < .05 in an unpaired Student's *t*-test. Abbreviations: CFSE, carboxyfluorescein succinimidyl ester; CsA, Cyclosporine A.

Flt3-L promotes myeloid and DC differentiation when added to serum-free suspension cultures of human CD34^+^ hematopoietic progenitor cells 49. We studied the effects of calcineurin inhibitor addition on the in vitro proliferation of Flt3-L-dependent human hematopoietic precursors. CD34^+^ progenitors were cultured in serum-free medium supplemented with Flt3-L in presence or absence of CsA for 3 days. CsA addition increased total cell number (Fig. 3A), percentage of dividing cells (Fig. 3B) as well as increased the rate of CFSE dye dilution (Fig. 3C). Therefore, CsA promotes the Flt3-dependent proliferation initiation of hematopoietic progenitor cells.

### Calcineurin-NFAT Inhibitors Selectively Increase GMP Proliferation In Vitro and Global Expression Profiling Reveals NFAT as a Cell Cycle Regulator in Progenitor Cells

**Figure 4:**
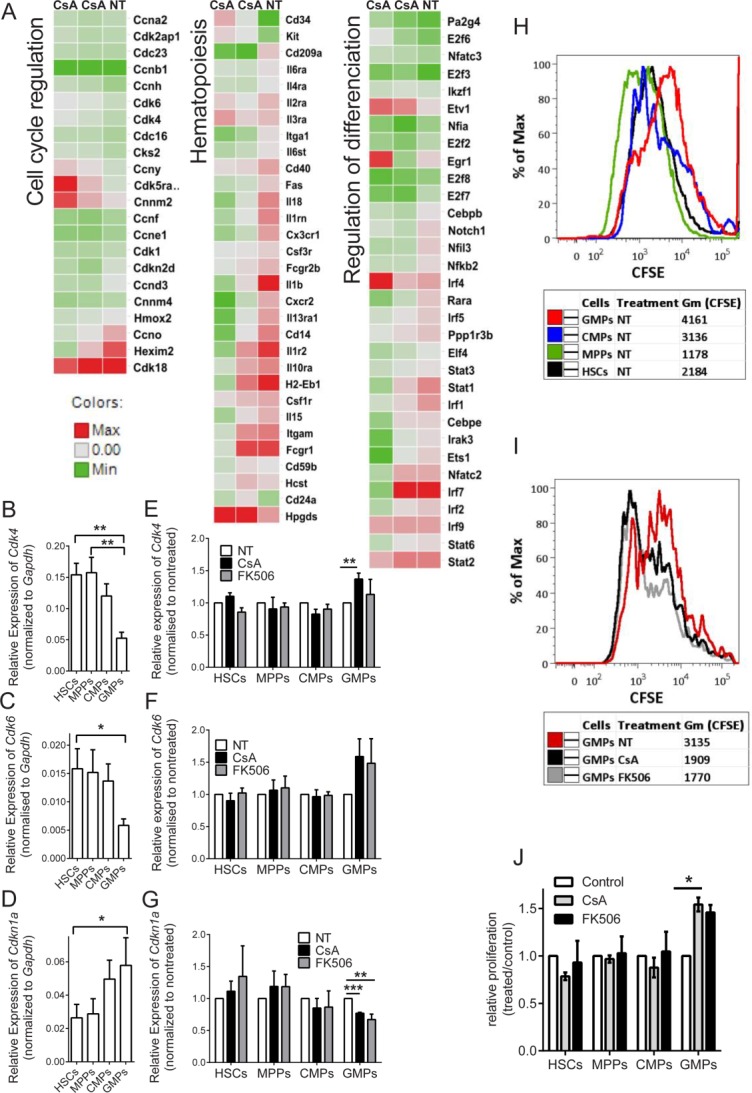
Calcineurin-nuclear factor of activated T cells (NFAT) inhibition results in increased granulocyte–monocyte progenitors (GMP) proliferation in vitro, due to changes in the expression of cell cycle control genes in FLT-3L-differentiated progenitors. (A): Heat maps reflecting changes in the expression of genes related to cell cycle regulation, transcriptional regulation of differentiation, and hematopoiesis are shown. cKIT^+^-enriched cells were cultured in hematopoietic stem cell (HSC) medium stimulated with of rmFlt3-L (300 ng/ml) in the presence or absence of Cyclosporine A (CsA) (24 and 48 hours). Data represent three replicates per time point and treatment. Cells from 10 mice were pooled for each biological replicate. (B–D): Differential expression of *Cdk4, Cdk6*, and *Cdkn1a* (*p21*) mRNA within progenitor populations. Sorted HSCs (lin^−^, cKit^+^, Sca-1^+^, CD34^+^, Flt3^−^), multipotent progenitors (MPPs) (lin^−^, cKit^+^, Sca-1^+^, CD34^+^, Flt3^+^), common myeloid progenitors (CMPs) (lin^−^, cKit^+^, Sca-1^−^, CD34^+^, CD16/32^−^), and GMPs (lin^−^, cKit^+^, Sca-1^−^, CD34^+^, CD16/32^+^) were cultured for 24 hours in HSC medium in the presence of Flt3-L (300 ng/ml) before the expression of main cell cycle regulatory genes was measured by qRT-PCR. Data represent at least five independent experiments, bone marrow (BM) from 15 mice pooled for each. One-way analysis of variance (ANOVA) with Turkey multiple comparisons test was used, *, *p* < .05; **, *p* < .01; and ***, *p* < .001. (E–G): Relative expression of *Cdk4, Cdk6*, and *Cdkn1a* (*p21*) mRNAs in progenitors in response to CsA or FK506 treatment. Sorted HSCs, MPPs, CMPs, and GMPs were cultured for 24 hours in HSC medium in the presence of CsA or FK506. Data represent at least three independent experiments. One-way ANOVA with Turkey multiple comparisons test was used. *, *p* < .05; **, *p* < .01 and ***, *p* < .001. (H, I): Progenitors were sorted into HSCs (lin^−^, cKit^+^, Sca-1^+^, CD34^+^, Flt3^−^), MPPs (lin^−^, cKit^+^, Sca-1^+^, CD34^+^, Flt3^+^), CMPs (lin^−^, cKit^+^, Sca-1^−^, CD34^+^, CD16/32^int^), and GMPs (lin^−^, cKit^+^, Sca-1^−^, CD34^+^, CD16/32^high^) and stained with CFSE. (H): Different proliferation rates of HSCs, MPPs, CMPs, and GMPs were assessed as CFSE dilution in 48 hours. (I): Differences in proliferation of GMPs treated in vitro with CsA or FK506. (J): Relative proliferation of HSCs (lin^−^, cKit^+^, Sca-1^+^, CD34^+^, Flt3^−^), MPPs (lin^−^, cKit^+^, Sca-1^+^, CD34^+^, Flt3^+^), CMPs (lin^−^, cKit^+^, Sca-1^−^, CD34^+^, CD16/32^int^), and GMPs (lin^−^, cKit^+^, Sca-1^−^, CD34^+^, CD16/32^high^), sorted and cultured in vitro in the presence or absence of calcineurin-NFAT inhibitors. Proliferation was assessed by bromodeoxyuridine (BrdU) staining. Representative of three independent experiments, mean ± SE is plotted, *n* = 3. *, *p* < .05 in an unpaired Student's *t*-test. Abbreviations: CFSE, carboxyfluorescein succinimidyl ester; CMP, common myeloid progenitors; CsA, Cyclosporine A; GMP, granulocyte–monocyte progenitor; HSC, hematopoietic stem cell; MPP, multipotent progenitors; NT, non-treated.

To identify the downstream processes activated by calcineurin-NFAT signaling during Flt3-L-driven differentiation of myeloid progenitors, we performed a global gene expression analysis on cKIT^+^-enriched lineage-negative primary BM cells. Pooled progenitor populations (predominantly HSCs, MPPs, CMPs, and GMPs) were cultured for 24 or 48 hours in HSC medium with Flt3-L, in the presence or absence of CsA or FK506. Differentially expressed genes (DEG) were then identified within the groups: regulation of cell cycle (GO:0051726), regulation of cell differentiation (GO:0045595), and hematopoiesis (GO:0030097) (Fig. 4A; Supporting Information Table 1). The GO processes and pathway enrichment analysis are shown (Supporting Information Fig. 4A, 4B). We detected increased expression of genes controlling the main cell cycle check points, as well as up-regulation of several genes responsible for myeloid cell differentiation (Fig. 4A).

To confirm the relevance of these trends in CsA treated patients, we have reanalyzed gene expression data comparing PBMCs collected of healthy donors and from stable kidney recipients under immunosuppressant monotherapy 50. The analysis showed significant activation of hematopoiesis and proliferation (Supporting Information Fig. 5A–5C). Furthermore, we compared our mouse array with the patient obtained data. Supporting Information Figure 6 shows that significant IPA processes induced with CsA treatment are similar in both mice and human cells.

Lineage-determining transcription factors were downregulated, suggesting a lower rate of differentiation in the presence of the inhibitors. In contrast, kinases including *Cdk4* and *Cdk6* were expressed at increased levels when the calcineurin-NFAT pathway was inhibited. To determine how calcineurin-NFAT inhibitor treatment affected transcription in different progenitor subpopulations, the expression of the DEGs identified by microarray analysis was measured in sorted HSCs, MPPs, CMPs, and GMPs cultured for 24 hours in HSC medium with Flt3-L in the presence or absence of CsA or FK506. The expression of the main kinases regulating the cell cycle G_0_ checkpoint, *Cdk4* and *Cdk6*, was significantly downregulated during differentiation toward GMPs (Fig. 4B, 4C). Conversely, expression of key inhibitors of cell cycle progression, including *Cdkn1a (p21)*, increased with differentiation toward GMPs (Fig. 4D). Comparable changes in *Cdk4, Cdk6*, and *Cdkn1a* (*p21*) expression were observed in the progenitors analyzed immediately after sorting (Supporting Information Fig. 7A–7C). The sorting strategy used and purity achieved is shown in Supporting Information Figure 1A, 1B. These data clearly suggested a decrease in the self-renewal rate of progenitors during the process of differentiation. Figure 4E, 4F show the relative changes in expression of *Cdk4* and *Cdk6* mRNAs in different progenitor populations following calcineurin-NFAT inhibition. *Cdk4* and *Cdk6* expression in GMPs remained significantly higher in the presence of inhibitors, and accordingly, expression of *Cdkn1a* (*p21*) was downregulated (Fig. 4G). This finding again indicated that GMPs are the sole progenitor target affected by CsA or FK506 treatment.

We next sorted HSCs, MPPs, CMPs, and GMPs from untreated mice and stimulated these cells with Flt3-L in vitro in the presence or absence of CsA or FK506 before assessing their proliferation by flow cytometry 1-2 days later using CFSE staining (Fig. 4H, 4I) or BrdU incorporation and DNA content analysis (Fig. 4J). In vitro, the progenitor populations showed different proliferation rates, with GMPs replicating the least when inhibitors were absent (Fig. 4H). In contrast, when CsA or FK506 was added, GMPs substantially increased their proliferation rate (Fig. 4I). GMPs exclusively responded to CsA and FK506 treatment by significantly increasing their proliferation rate relative to GMPs in control cultures (Fig. 4J). Total cell numbers from these cultured progenitors show similar trends (Supporting Information Fig. 2E).

### Myeloid Progenitors Express Functional NFAT

**Figure 5:**
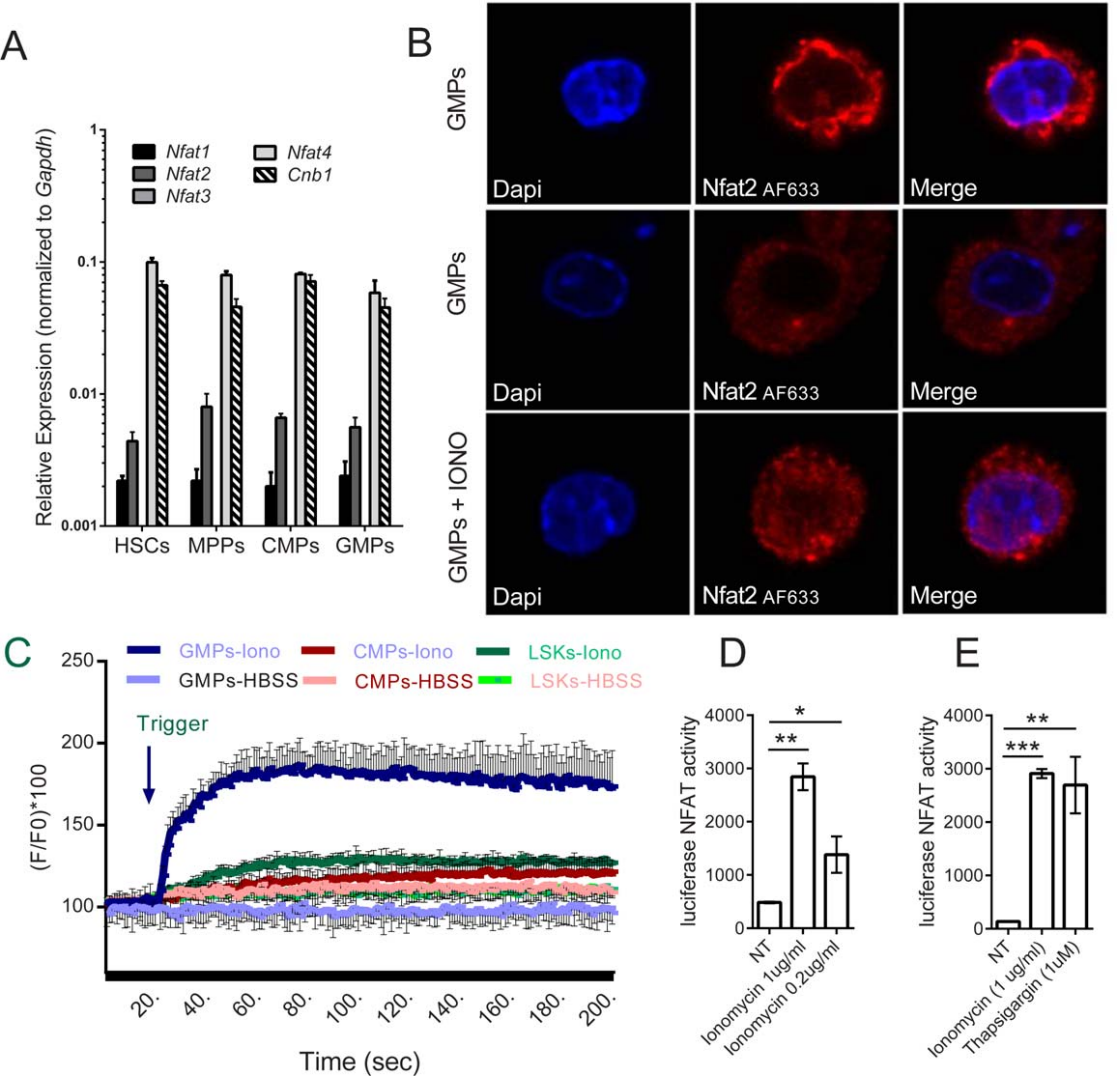
The calcineurin-nuclear factor of activated T cells (NFAT) pathway is present and functional in myeloid progenitors. (A): Quantitative polymerase chain reaction analysis of *Nfat1-4* and calcineurin *Cnb1* mRNA expression in hematopoietic stem cells (HSCs), multipotent progenitors (MPPs), common myeloid progenitors (CMPs), and granulocyte–monocyte progenitors (GMPs). Data are presented as mean ± SE from at least three independent experiments, in which bone marrow (BM) cells from at least 15 mice were pooled for sorting. Gating strategy used for flow cytometric sorting of subpopulations of hematopoietic progenitors identified as HSCs (lin^−^, cKit^+^, Sca-1^+^, CD34^+^, Flt3^−^), MPPs (lin^−^, cKit^+^, Sca-1^+^, CD34^+^, Flt3^+^), CMPs (lin^−^, cKit^+^, Sca-1^−^, CD34^+^, CD16/32^int^), and GMPs (lin^−^, cKit^+^, Sca-1^−^, CD34^+^, CD16/32^high^) is shown at Supporting Information Figure 1. (B): Representative confocal images of sorted GMPs. GMPs were incubated for 1 hour (37°C) with anti-NFAT2 antibody (Thermo Scientific) diluted to 10 µg/ml in the blocking solution, washed three times in phosphate-buffered saline and incubated for 1 hour (37°C) with the secondary antibody (AlexaFluor 633 goat anti-mouse IgG, Molecular Probes) and nuclei were stained with DAPI (original magnification, x200). Images were captured from cells labeled immediately after sorting (first line) or after 24 hours culture in HSC medium (lines 2 and 3). Ionomycin (1 μg/ml for 15 minutes) was used to induce NFAT nuclear translocation. Cellular localization of NFAT2 was visualized using an Olympus FV1000 confocal microscope. (C): Representative graph of fluorometric Ca^2+^ mobilization analysis in sorted BM progenitors (LSKs, CMPs, and GMPs). Ionomycin (500 ng/ml) or HBSS were added after 20 seconds of measurement. BM cells from at least 15 mice were pooled for sorting. (D, E): Cells were transduced with a NFAT responsive element luciferase reporter construct. NFAT translocated to the nucleus and started transcription of the reporter gene upon ionomycin stimulation of the HSC line (D) and in lineage-depleted, cKIT^+^ purified progenitor cells (E). Data are presented as mean ±SE from one of three independent experiments. *, *p* < .05; **, *p* < .01 and ***, *p* < .001 in an unpaired Student's *t*-test. Abbreviations: CMP, common myeloid progenitors; DAPI, 4′,6-diamidino-2-phenylindole; GMP, granulocyte–monocyte progenitor; HBSS, Hanks' balanced salt solution; HSC, hematopoietic stem cell; IONO, ionomycin; LSK, lin^−^Sca1^+^cKIT^+^ bone marrow cells; MPP, multipotent progenitors; NFAT, nuclear factor of activated T cells; NT, non-treated.

We next determined whether *Nfat1-4* were expressed at the mRNA level in sorted hematopoietic progenitor cell populations of HSCs, MPPs, CMPs, and GMPs. Progenitors were isolated from lineage-depleted BM cells according to the gating strategy shown in Supporting Information Fig. 1. mRNA expression levels of NFAT family members were measured after 24 hours of culture in HSC medium (Fig. 4A). Each progenitor population expressed *Nfat1, 2, 4*, and *Cnb1* (Fig. 5A), which was confirmed in cells analyzed immediately after sorting (Supporting Information Fig. 7A, 7B). Expression of Nfat2 protein in GMPs was confirmed by confocal microscopy (Fig. 5B); Partial nuclear translocation of Nfat2 protein occurred after ionomycin-triggered Ca^2+^ release, indicating that Nfat2 was able to respond functionally in these cells (Fig. 5B). However, treatment with ionomycin (which mobilizes calcium from intracellular stores) resulted in variable levels of intracellular Ca^2+^ increase across the different progenitor populations (Fig. 5C; Supporting Information Fig. 9A): increases in Ca^2+^ levels in GMPs were substantially higher than in CMPs and LSKs. This led us to investigate NFAT functionality in more detail within the myeloid lineage using a NFAT luciferase reporter prepared from a previously established HSC line 51. Treating HSCs with ionomycin or thapsigargin (which inhibits intracellular Ca^2+^ clearance) confirmed NFAT expression and translocation to the nuclei with consequent dose-dependent induction of luciferase transcription (Fig. 5D). These results were replicated in primary cKIT^+^-enriched, lineage-negative cells isolated from BM and transduced with the NFAT reporter construct (Fig. 5E). Taken together these data demonstrate the presence and functionality of the Ca^2+^-calcineurin-NFAT pathway at early stages of differentiation of myeloid progenitors.

### Flt3-L Mediates Activation of the Calcineurin-NFAT Pathway in GMPs

**Figure 6:**
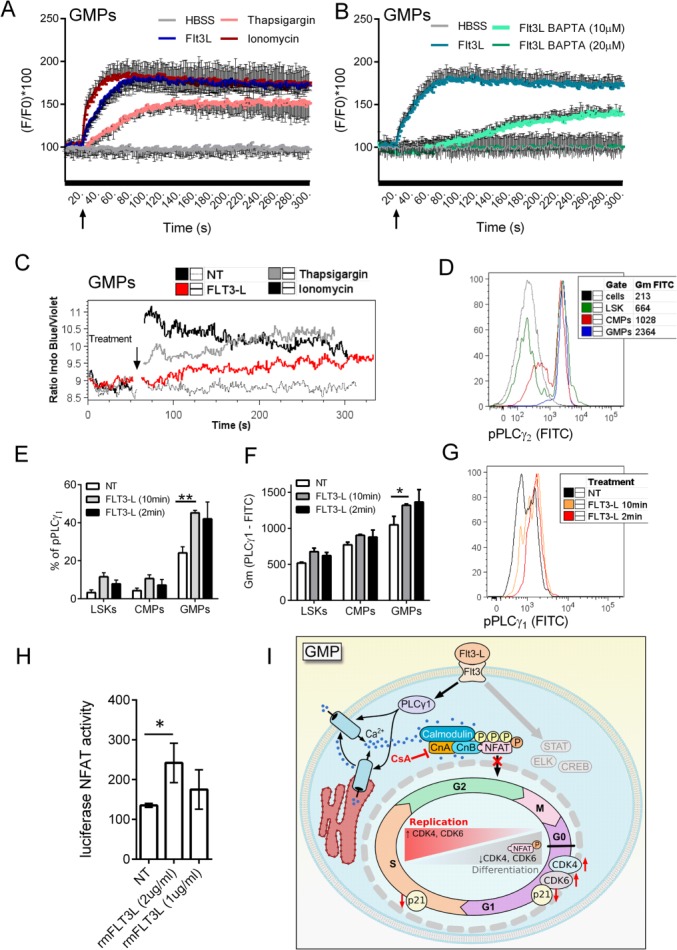
Flt3-L triggers signaling leading to nuclear factor of activated T cells (NFAT) translocation in granulocyte–monocyte progenitors (GMPs). (A): Levels of intracellular Ca^2+^ measured as Fluo4 fluorescence in sorted lin^−^Sca1^+^cKIT^+^ bone marrow (BM) cells (LSKs), common myeloid progenitors (CMPs), and GMPs. After 20 seconds of measurements cells were triggered with FLT3-L, ionomycin, thapsigargin, or Hanks' balanced salt solution (HBSS), and Ca^2+^ levels were assessed for another 5 minutes. (B): Intracellular Ca^2+^ chelator BAPTA was used to block Ca^2+^ release induced by FLT3-L in sorted GMPs. (C): Flt3-L induces Ca^2+^ release in GMPs sorted as lin^−^, cKit^+^, Sca-1^−^, CD34^+^, CD16/32^high^. Graph of Ca^2+^ release analysis in GMPs from lineage-depleted BM cells. Flt3-L (1μg/ml) was added after 1 minute of measurement followed by 4 minutes Ca^2+^ release measurement. Representative of three independent experiments, where BM cells from five mice were pooled. (D): Different levels of phospholipase Cγ (PLCγ_2_) phosphorylation (pPLCγ_2_) in LSKs (pool of hematopoietic stem cell [HSCs] and multipotent progenitors [MPPs]; lin^−^, cKit^+^, Sca-1^+^), CMPs, and GMPs in freshly isolated BM. (E–G): Different levels of pPLCγ_1_ in progenitor populations before and after Flt3-L administration. Lineage-depleted BM cells were cultured for 4 hours in HSC medium, Flt3-L (1 μg/ml) was added 15 minutes before fixing and labeling with antibodies for progenitor markers, PLCγ_1_ and pPLCγ_2_. Cells were gated as LSKs (pool of HSCs and MPPs; lin^−^, cKit^+^, Sca-1^+^), CMPs (lin^−^, cKit^+^, Sca-1^−^, CD34^+^, CD16/32^int^), and GMPs (lin^−^, cKit^+^, Sca-1^−^, CD34^+^, CD16/32^high^). (E, F): Percentage of pPLCγ_1_ cells and Gm of fluorescence of pPLCγ_1_ and double labeling of total PLCγ_1_ with pPLCγ_1_ upon trigger with Flt3-L are shown. Data are presented as mean ± SE, *, *p* < .05 and **, *p* < .01 in an unpaired Student's *t*-test. (G): Histogram overlay of pPLCγ_1_ intensity in GMPs after the Flt3-L trigger. Representative experiment from three independent experiments is shown. (H): Flt3-L induces NFAT translocation in cKIT^+^-enriched BM cells. BM cells were depleted of lineaged cells and enriched with cKIT^+^ beads, maintained in HSC medium and transduced with NFAT reporter constructs. Transfected cells were kept in HSC medium for 48 hours, and 1 μg/ml of Flt3-L was added for last 4 hours of culture before nuclear translocation of NFAT reflecting activity of luciferase reporter gene was measured. Data are presented as mean ± SE from one representative of three independent experiments, *, *p* < .05 in an unpaired Student's *t*-test. BM pooled from 10 mice was used for each experiment. (I): Proposed graphical scheme of cell cycle regulation in GMPs. Flt3-L activates PLCγ_1_ and induce increase in intracellular Ca^2+^ levels, this further activates calcineurin and NFAT translocation. When calcineurin-NFAT interaction is blocked, expression of cell cycle regulation genes in GMPs is changed to promote further proliferation. Abbreviations: CMP, common myeloid progenitors; CsA, Cyclosporine A; FITC, fluorescein isothiocyanate; GMP, granulocyte–monocyte progenitor; HBSS, Hanks' balanced salt solution; LSK, lin^−^Sca1^+^cKIT^+^ bone marrow cells; NFAT, nuclear factor of activated T cells; NT, non-treated; PLC, phospholipase Cγ.

Since calcineurin and NFAT members are expressed in multiple different hematopoietic progenitors, we next assessed whether the main myeloid growth factor Flt3-L might be involved in triggering calcineurin-NFAT signaling to regulate the cell cycle and proliferation rate of these populations. LSKs, CMPs, and GMPs were sorted from BM and loaded with Fluo4-NW, before being stimulated with Flt3-L, ionomycin, or thapsigargin and assessed for changes in intracellular Ca^2+^ levels using a spectrophotometer (Fig. 6A). We observed that Flt3-L-induced Ca^2+^ release was effectively blocked by addition of Ca^2+^ chelator BAPTA (Fig. 6B). We also confirmed our findings by using flow-cytometry to identify changes in intracellular Ca^2+^ levels in different populations of BM progenitors stained, loaded with INDO-1 and exposed to Flt3-L (Fig. 6C). In agreement with the high sensitivity of GMPs to intracellular Ca^2+^ release induced by ionomycin treatment (Fig. 5C; Supporting Information Fig. 9A), GMPs also displayed marked increases in intracellular Ca^2+^ levels upon Flt3-L stimulation (Fig. 6A, 6C), while LSKs and CMPs did not increase the levels of intracellular Ca^2+^ upon Flt3-L stimulation (Supporting Information Fig. 9B). Flt3-L, which is expressed on GMPs as well as MPPs and CMPs (Supporting Information Fig. 9C), is associated with PLCγ as a possible inducer of Ca^2+^ entry; so we next measured expression and phosporylation of PLCγ_1_ and PLCγ_2_ in myeloid cell progenitors (Fig. 6D–6G). Steady-state phosphorylation of PLCγ_1_(pPLCγ_1_) was observed to increase in parallel with cell differentiation from Lin^−^, Sca-1^+^, c-Kit^+^ progenitors (pooled HSCs and MPPs, referred to as LSK) to GMPs (Fig. 6E–6G) and was further significantly elevated in GMPs by 10 minutes stimulation with Flt3-L (Fig. 6E–6G; Supporting Information Fig. 9D). On contrary, PLCγ_2_ seems to be heavily phosphorylated in steady state in freshly isolated GMPs (Fig. 6D), and we have not observed increase in phosphorylation after FLT3-L trigger (Supporting Information Fig. 9E, 9F). To confirm that Flt3-L stimulation results in NFAT translocation in myeloid progenitors, lineage-depleted, cKIT^+^-enriched cells were isolated from BM and immediately transduced with the NFAT luciferase reporter construct. Treatment with Flt3-L resulted in significantly increased luciferase activity, thereby reflecting functional NFAT stimulation (Fig. 6H).

In summary, we demonstrate that Flt3-L mobilized Ca^2+^ in GMPs and increased phosphorylation of PLCγ_1_. Moreover, primary cKIT^+^-enriched BM cells transduced with an NFAT reporter gene confirmed functional translocation of NFAT upon Flt3-L stimulation. Taken together, these findings show that GMP cell cycle regulation is regulated by Flt3-L activation of PLCγ_1_ and induction of Ca^2+^ entry, which in-turn activates calcineurin and NFAT translocation. Calcineurin-NFAT signaling subsequently modulates the expression of genes that affect the cell cycle progression of GMPs so that these cells can proliferate more rapidly than other progenitor populations (Fig. 6I).

## Discussion

In the current report, we identified and characterized a new pathway that regulates the cell cycle specifically in GMPs (Fig. 6I). In vivo treatment with CsA or FK506 facilitated the cycling of GMPs, leading to a rapid increase in GMP numbers in the BM. Conditional Cnb1-knockout mice were used to confirm these findings by mixing BM cells from control and Cnb1-knockout mice and engrafting them into irradiated recipients. Soon after knockout induction with polyI:C, the majority of GMPs was observed to originate from the calcineurin-impaired donor cells, while the ratio of CMPs remained as injected. These data indicate a specific in vivo effect of calcineurin-NFAT signaling on GMPs proliferative potential. Consistent with these data, sorted, GMPs proliferated less than HSCs, MPPs and CMPs when left untreated during in vitro culture, but addition of CsA or FK506 to these cultures was sufficient to significantly increase GMP proliferation.

To dissect the mechanism of enhanced GMP proliferation under NFAT inhibition, we performed a microarray analysis of gene expression on progenitor cells following brief differentiation with Flt3-L in the presence of calcineurin-NFAT inhibitors. We observed similar changes in PBMCs from CsA treated patients. Global gene expression analysis from mouse BM progenitors revealed marked changes in expression of cell cycle regulation genes as a result of CsA treatment, later validated specifically in GMPs. An important observation was the gradual downregulation of *Cdk4* and *Cdk6* and upregulation of *Cdkn1a* (*p21*) gene expression with the differentiation of cells from HSCs through MPPs, CMPs, and GMPs. This suggests a link between cell differentiation and progression through the cell cycle, mediated by NFAT and triggered by Flt3-L. Our results indicate that the process of slowing down the cell cycle during differentiation is perturbed by calcineurin-NFAT inhibition, particularly at the level of GMPs, both in vitro and in vivo. Therefore, we conclude that the calcineurin-NFAT pathway plays a key role in inhibiting GMP proliferation to regulate myeloid cell differentiation. Our results are supported by other studies in which cell cycle gene regulation was suggested to be important for HSC quiescence 52. Furthermore, excessive progenitor proliferation led to the exhaustion of HSCs 53,54, which has recently been linked to NFAT signaling 14. Several cell cycle-regulating genes including cyclin D1 55, cyclin A2 56, *Cdk4* 20, and *Cdk6* 55,57 are repressed by NFAT in multiple different cell types, and NFAT1-deficient mice exhibit increased levels of Cdk4 58. Furthermore, inhibitors of the cell cycle such as Cdkn1a (p21) and Cdk2 are also NFAT dependent 59,60. These master cell cycle regulators are the main controllers of differentiation during early hematopoietic events as well as in the progression from CMPs to GMPs 22,52,61.

Here, we provide evidence that *Nfat*1, 2, 4, and calcineurin (*Cnb1*) are ubiquitously expressed in murine HSCs, MPPs, CMPs, and GMPs. Similarly, others have reported NFAT expression in human CD34^+^ immunomobilized progenitors, which hinted at a potential role in myeloid differentiation 15. We show for the first time that GMPs are exquisitely sensitive to induced Ca^2+^ release which is required for NFAT activation. Furthermore, NFAT was efficiently translocated to the nucleus following activation in cKit^+^-enriched progenitors. Thus, the calcineurin-NFAT pathway is both present and functional in hematopoietic progenitors, especially in GMPs. Several studies have aimed to assess the role of calcineurin-NFAT signaling in myeloid development: Gallo et al. observed small and nonsignificant increases in myeloid cell numbers upon conditional KO induction of Cnb1 in HSCs, concluding that Cnb1 is not necessary for development of the myeloid compartment 13. In a different experimental setting, we found that progenitors expressing the VIVIT peptide inhibitor of calcineurin-NFAT 62, give rise to increased numbers of myeloid cells. DCs and CD11b^+^Gr1^+^ myeloid cells derived from calcineurin-NFAT-impaired progenitors possessed a substantial developmental advantage over their control counterparts when engrafted into irradiated mice 1. Similarly, increased numbers of GM-CFU were shown when enriched human CD34^+^ progenitors were treated with FK506 in vitro 63. Congruent with these observations, we here demonstrated that CsA promotes Flt3-L-dependent initial cell proliferation of purified human CD34^+^ progenitor cells.

Notably, we illustrated that the calcineurin-NFAT pathway in GMPs is triggered by Flt3-L through pPLCγ_1_ and Ca^2+^ release, thus revealing another mechanism by which this growth factor regulates myeloid development. PLCγ_1_ phosphorylation followed by NFAT signaling has been shown to be essential in T cell development, activation, and survival 64, while other growth factors such as G-, M-, and GM-CSF regulate lineage commitment through PLCγ_2_ 25,30,31. The source of Ca^2+^ driving these processes remains to be elucidated, since PLCγ can induce both exogenous flux as well and endogenous release 65. The relative contributions made by PLCγ_1_ and PLCγ_2_ to intracellular or extracellular Ca^2+^ entry will also require further analysis, since cooperation of Ca^2+^ entry from both compartments is particularly important for NFAT translocation 4,66.

Flt3-L-induced NFAT signaling leads to cell cycle progression in GMPs that is controlled via a coordinated program of NFAT-regulated changes in expression of cell cycle control genes. Possible roles for other NFAT binding partners have yet to be investigated. In this study, we show that activation of GMPs with Flt3-L induces phosphorylation of PLCγ_1_ and consequently stimulates intracellular Ca^2+^ release, which does not occur in HSCs, MPPs or CMPs. This Ca^2+^ release initiates translocation of Nfat2 to the nucleus and the transcription of target genes. In addition, we showed that the frequency of cells expressing pPLCγ_1_, the main regulator of store-operated Ca^2+^ release, is increased in parallel with progenitor differentiation. The role played by Flt3 signaling in normal hematopoiesis was previously thought to be mediated solely by activation of Stat5, RAS/MAPK and PI3K 67. However, deregulation of Flt3 signaling by activating mutation is present in one third of acute myeloid leukemia cases where expansion of GMPs occurs 26,32. The role of pPLCγ_1_, Ca^2+^, and NFAT signaling with respect to Flt3 signaling in this disease is currently unknown.

Ca^2+^ signaling in immune cells is known to have two qualitatively different outcomes: a short peak of Ca^2+^ release results in immunological synapse formation and granule exocytosis, while the type of prolonged Ca^2+^ signaling induced by growth factors or other cytokines has been shown to enhance NFAT-dependent transcription. This has been reported in T cells 68,69 and also during embryonic development 70. The regulatory role of Flt3 in steady state development of GMPs has been suggested 43, leading us to hypothesize that this regulation might involve the NFAT pathway. Flt3 directly regulates HSC quiescence and homeostasis, 67,70 as well as DC development 33–36. Flt3 is also expressed in highly proliferating MPPs 22,71,72 and the more differentiated CMPs and GMPs 36,37,43. The role of Flt3 signaling is clearly linked to regulation of hematopoietic progenitor numbers, as shown in both Flt3 and Flt3-L KO models 34,39, but to date, the involvement of Flt3 in the early events of steady state hematopoiesis has not been fully appreciated. Further support for our results comes from the finding that an activating mutation in *Flt3* leads to the development of a myeloproliferative disorder 73 that is characterized by increased numbers of GMPs and an accumulation of mature myeloid cells 74–76.

## Conclusions

Our data reveal a novel role for Flt3 signaling in NFAT activation and regulation of myelopoiesis. Investigation of the underlying mechanisms by which NFAT inhibition can increase myelopoiesis uncovered direct regulation of cell cycle control genes *Cdk4, Cdk6*, and *Cdkn1a* (*p21*) by NFAT, specifically in GMPs (Fig. 5I). The calcineurin-NFAT pathway is a therapeutic target in multiple conditions including donor organ rejection, graft-versus-host disease, and autoimmune disorders. While T cells are the main cell type known to be subject to CsA and FK506 immunosuppression, here we provide evidence of an important influence of these drugs on myeloid cell hematopoiesis via direct effects on GMP proliferation. Clearly, the full range of effects these drugs exert on the immune system is not fully appreciated, though the prevalence of side effects following their administration is driving new research into their mechanisms of action. Improved knowledge of the roles played by NFAT during myeloid hematopoiesis will provide insight into clinical studies aiming to better understand homeostatic regulation.

## Acknowledgments

We thank J. Lum for microarray processing. C. Phua and S. Nabti for animal handling; I. Low and N. Shadan from SIgN flow cytometry facility for cell sorting; H.S. Tay for technical assistance; K. Karjalainen for HSC lines; and L. Robinson with N. McCarthy from Insight Editing London for review of the paper, A. Wong for the graphical abstract. This work was supported by the BMRC, A*STAR, Singapore.

## Author Contributions

J.F.: designed and conducted the majority of the experiments, analyzed data and wrote the manuscript; C.X.F.L., A.M., and E.V.: designed, performed, and analyzed some experiments; B.T.K.L. and J.C.: performed microarray data analysis; T.Z.: designed and analyzed some experiments and revised the manuscript; A.L.: supervised flow cytometry and sorting experiments; F.Z. and M.P.: supervised microarray processing and analysis; H.S.: supervised some experiments and revised the manuscript; P.R.C.: supervised the project and revised the manuscript. A.M. and C.X.F.L. are joint second authors.

## Disclosure of Potential Conflicts of Interest

The authors indicate no potential conflicts of interest.

## Supporting Information

See www.StemCells.com for supporting information available online.

## References

[1] Fric J, Lim CX, Koh EG (2012). Calcineurin/NFAT signalling inhibits myeloid haematopoiesis. EMBO Mol Med.

[2] Fric J, Zelante T, Wong AY (2012). NFAT control of innate immunity. Blood.

[3] Macian F (2005). NFAT proteins: Key regulators of T-cell development and function. Nat Rev Immunol.

[4] Hogan PG, Chen L, Nardone J (2003). Transcriptional regulation by calcium, calcineurin, and NFAT. Genes Dev.

[5] Feske S, Okamura H, Hogan PG (2003). Ca2+/calcineurin signalling in cells of the immune system. Biochem Biophys Res Commun.

[6] Graef I, Chen F, Chen L (2001). Signals transduced by Ca(2+)/calcineurin and NFATc3/c4 pattern the developing vasculature. Cell.

[7] de la Pompa J, Timmerman L, Takimoto H (1998). Role of the NF-ATc transcription factor in morphogenesis of cardiac valves and septum. Nature.

[8] Zelante T, Fric J, Wong AY (2012). Interleukin-2 production by dendritic cells and its immuno-regulatory functions. Front Immunol.

[9] Zanoni I, Ostuni R, Capuano G (2009). CD14 regulates the dendritic cell life cycle after LPS exposure through NFAT activation. Nature.

[10] Greenblatt MB, Aliprantis A, Hu B (2010). Calcineurin regulates innate antifungal immunity in neutrophils. J Exp Med.

[11] Buxade M, Lunazzi G, Minguillon J (2012). Gene expression induced by Toll-like receptors in macrophages requires the transcription factor NFAT5. J Exp Med.

[12] Zaslavsky A, Chou ST, Schadler K (2013). The calcineurin-NFAT pathway negatively regulates megakaryopoiesis. Blood.

[13] Gallo EM, Ho L, Winslow MM (2008). Selective role of calcineurin in haematopoiesis and lymphopoiesis. EMBO Rep.

[14] Bauer W, Rauner M, Haase M (2011). Osteomyelosclerosis, anemia and extramedullary hematopoiesis in mice lacking the transcription factor NFATc2. Haematologica.

[15] Kiani A, Habermann I, Haase M (2004). Expression and regulation of NFAT (nuclear factors of activated T cells) in human CD34+ cells: Down-regulation upon myeloid differentiation. J Leukoc Biol.

[16] Boyer S, Schroeder A, Smith-Berdan S (2011). All hematopoietic cells develop from hematopoietic stem cells through Flk2/Flt3-positive progenitor cells. Cell Stem Cell.

[17] Kiani A, Kuithan H, Kuithan F (2007). Expression analysis of nuclear factor of activated T cells (NFAT) during myeloid differentiation of CD34+ cells: Regulation of Fas ligand gene expression in megakaryocytes. Exp Hematol.

[18] Negishi-Koga T, Takayanagi H (2009). Ca2+-NFATc1 signaling is an essential axis of osteoclast differentiation. Immunol Rev.

[19] Caetano M, Vieira-de-Abreu A, Teixeira L (2002). NFATC2 transcription factor regulates cell cycle progression during lymphocyte activation: Evidence of its involvement in the control of cyclin gene expression. Faseb J.

[20] Horsley V, Aliprantis AO, Polak L (2008). NFATc1 balances quiescence and proliferation of skin stem cells. Cell.

[21] Li X, Zhu L, Yang A (2011). Calcineurin-NFAT signaling critically regulates early lineage specification in mouse embryonic stem cells and embryos. Cell Stem Cell.

[22] Passegué E, Wagers A, Giuriato S (2005). Global analysis of proliferation and cell cycle gene expression in the regulation of hematopoietic stem and progenitor cell fates. J Exp Med.

[23] Majumdar MK, Thiede MA, Mosca JD (1998). Phenotypic and functional comparison of cultures of marrow-derived mesenchymal stem cells (MSCs) and stromal cells. J Cell Physiol.

[24] Baldridge M, King K, Goodell M (2011). Inflammatory signals regulate hematopoietic stem cells. Trends Immunol.

[25] Jack GD, Zhang L, Friedman AD (2009). M-CSF elevates c-Fos and phospho-C/EBPalpha(S21) via ERK whereas G-CSF stimulates SHP2 phosphorylation in marrow progenitors to contribute to myeloid lineage specification. Blood.

[26] Gilliland DG, Griffin JD (2002). The roles of FLT3 in hematopoiesis and leukemia. Blood.

[27] Adolfsson J, Månsson R, Buza-Vidas N (2005). Identification of Flt3+ lympho-myeloid stem cells lacking erythro-megakaryocytic potential a revised road map for adult blood lineage commitment. Cell.

[28] Paredes-Gamero EJ, Leon CM, Borojevic R (2008). Changes in intracellular Ca2+ levels induced by cytokines and P2 agonists differentially modulate proliferation or commitment with macrophage differentiation in murine hematopoietic cells. J Biol Chem.

[29] Barbosa CM, Leon CM, Nogueira-Pedro A (2011). Differentiation of hematopoietic stem cell and myeloid populations by ATP is modulated by cytokines. Cell Death Dis.

[30] Leon CM, Barbosa C, Justo G (2011). Requirement for PLCγ2 in IL-3 and GM-CSF-stimulated MEK/ERK phosphorylation in murine and human hematopoietic stem/progenitor cells. J Cell Physiol.

[31] Barbosa CM, Bincoletto C, Barros CC (2014). PLCgamma2 and PKC are important to myeloid lineage commitment triggered by M-SCF and G-CSF. J Cell Biochem.

[32] Choudhary C, Müller-Tidow C, Berdel W (2005). Signal transduction of oncogenic Flt3. Int J Hematol.

[33] Waskow C, Liu K, Darrasse-Jèze G (2008). The receptor tyrosine kinase Flt3 is required for dendritic cell development in peripheral lymphoid tissues. Nat Immunol.

[34] McKenna HJ, Stocking KL, Miller RE (2000). Mice lacking flt3 ligand have deficient hematopoiesis affecting hematopoietic progenitor cells, dendritic cells, and natural killer cells. Blood.

[35] Onai N, Obata-Onai A, Tussiwand R (2006). Activation of the Flt3 signal transduction cascade rescues and enhances type I interferon-producing and dendritic cell development. J Exp Med.

[36] D'Amico A, Wu L (2003). The early progenitors of mouse dendritic cells and plasmacytoid predendritic cells are within the bone marrow hemopoietic precursors expressing Flt3. J Exp Med.

[37] Karsunky H, Merad M, Cozzio A (2003). Flt3 ligand regulates dendritic cell development from Flt3+ lymphoid and myeloid-committed progenitors to Flt3+ dendritic cells in vivo. J Exp Med.

[38] Matthews W, Jordan C, Wiegand G (1991). A receptor tyrosine kinase specific to hematopoietic stem and progenitor cell-enriched populations. Cell.

[39] Mackarehtschian K, Hardin J, Moore K (1995). Targeted disruption of the flk2/flt3 gene leads to deficiencies in primitive hematopoietic progenitors. Immunity.

[40] Hudak S, Hunte B, Culpepper J (1995). FLT3/FLK2 ligand promotes the growth of murine stem cells and the expansion of colony-forming cells and spleen colony-forming units. Blood.

[41] Liu K, Nussenzweig M (2010). Origin and development of dendritic cells. Immunol Rev.

[42] Guermonprez P, Helft J, Claser C (2013). Inflammatory Flt3l is essential to mobilize dendritic cells and for T cell responses during Plasmodium infection. Nat Med.

[43] Böiers C, Buza-Vidas N, Jensen C (2010). Expression and role of FLT3 in regulation of the earliest stage of normal granulocyte-monocyte progenitor development. Blood.

[44] Yasmin N, Bauer T, Modak M (2013). Identification of bone morphogenetic protein 7 (BMP7) as an instructive factor for human epidermal Langerhans cell differentiation. J Exp Med.

[45] Taschner S, Koesters C, Platzer B (2007). Down-regulation of RXRalpha expression is essential for neutrophil development from granulocyte/monocyte progenitors. Blood.

[46] Crabtree GR (1999). Generic signals and specific outcomes: Signaling through Ca2+, calcineurin, and NF-AT. Cell.

[47] Zeng H, Chattarji S, Barbarosie M (2001). Forebrain-specific calcineurin knockout selectively impairs bidirectional synaptic plasticity and working/episodic-like memory. Cell.

[48] Kuhn R, Schwenk F, Aguet M (1995). Inducible gene targeting in mice. Science.

[49] Strobl H, Bello-Fernandez C, Riedl E (1997). flt3 ligand in cooperation with transforming growth factor-beta1 potentiates in vitro development of Langerhans-type dendritic cells and allows single-cell dendritic cell cluster formation under serum-free conditions. Blood.

[50] Brouard S, Puig-Pey I, Lozano JJ (2010). Comparative transcriptional and phenotypic peripheral blood analysis of kidney recipients under cyclosporin A or sirolimus monotherapy. Am J Transplant.

[51] Ruedl C, Khameneh HJ, Karjalainen K (2008). Manipulation of immune system via immortal bone marrow stem cells. Int Immunol.

[52] Matsumoto A, Takeishi S, Kanie T (2011). p57 is required for quiescence and maintenance of adult hematopoietic stem cells. Cell Stem Cell.

[53] Akala OO, Clarke MF (2006). Hematopoietic stem cell self-renewal. Curr Opin Genet Dev.

[54] Orford K, Scadden D (2008). Deconstructing stem cell self-renewal: Genetic insights into cell-cycle regulation. Nat Rev Genet.

[55] Karpurapu M, Wang D, Van Quyen D (2010). Cyclin D1 is a bona fide target gene of NFATc1 and is sufficient in the mediation of injury-induced vascular wall remodeling. J Biol Chem.

[56] Carvalho L, Teixeira L, Carrossini N (2007). The NFAT1 transcription factor is a repressor of cyclin A2 gene expression. Cell Cycle.

[57] Singh NK, Kundumani-Sridharan V, Kumar S (2012). Protein kinase N1 is a novel substrate of NFATc1-mediated cyclin D1-CDK6 activity and modulates vascular smooth muscle cell division and migration leading to inward blood vessel wall remodeling. J Biol Chem.

[58] Baksh S, Widlund HR, Frazer-Abel AA (2002). NFATc2-mediated repression of cyclin-dependent kinase 4 expression. Mol Cell.

[59] Santini MP, Talora C, Seki T (2001). Cross talk among calcineurin, Sp1/Sp3, and NFAT in control of p21(WAF1/CIP1) expression in keratinocyte differentiation. Proc Natl Acad Sci U S A.

[60] Gallo EM, Winslow MM, Cante-Barrett K (2007). Calcineurin sets the bandwidth for discrimination of signals during thymocyte development. Nature.

[61] Rosu-Myles M, Taylor B, Wolff L (2007). Loss of the tumor suppressor p15Ink4b enhances myeloid progenitor formation from common myeloid progenitors. Exp Hematol.

[62] Aramburu J, Garcia-Cozar F, Raghavan A (1998). Selective inhibition of NFAT activation by a peptide spanning the calcineurin targeting site of NFAT. Mol Cell.

[63] Hirao A, Kawano Y, Takaue Y (1993). Effects of immunosuppressants, FK506, deoxyspergualin, and cyclosporine A on immature human hematopoiesis. Blood.

[64] Yamane H, Paul WE (2013). Early signaling events that underlie fate decisions of naive CD4(+) T cells toward distinct T-helper cell subsets. Immunol Rev.

[65] Patterson RL, van Rossum DB, Nikolaidis N (2005). Phospholipase C-gamma: Diverse roles in receptor-mediated calcium signaling. Trends Biochem Sci.

[66] Sharma S, Quintana A, Findlay GM (2013). An siRNA screen for NFAT activation identifies septins as coordinators of store-operated Ca2+ entry. Nature.

[67] Chu S, Heiser D, Li L (2012). FLT3-ITD Knockin Impairs Hematopoietic Stem Cell Quiescence/Homeostasis, Leading to Myeloproliferative Neoplasm. Cell Stem Cell.

[68] Feske S (2007). Calcium signalling in lymphocyte activation and disease. Nat Rev Immunol.

[69] Dolmetsch R, Lewis R, Goodnow C (1997). Differential activation of transcription factors induced by Ca2+ response amplitude and duration. Nature.

[70] Muller MR, Sasaki Y, Stevanovic I (2009). Requirement for balanced Ca/NFAT signaling in hematopoietic and embryonic development. Proc Natl Acad Sci U S A.

[71] Adolfsson J, Borge O, Bryder D (2001). Upregulation of Flt3 expression within the bone marrow Lin(-)Sca1(+)c-kit(+) stem cell compartment is accompanied by loss of self-renewal capacity. Immunity.

[72] Christensen J, Weissman I (2001). Flk-2 is a marker in hematopoietic stem cell differentiation: A simple method to isolate long-term stem cells. Proc Natl Acad Sci U S A.

[73] Kiyoi H, Towatari M, Yokota S (1998). Internal tandem duplication of the FLT3 gene is a novel modality of elongation mutation which causes constitutive activation of the product. Leukemia.

[74] Lee B, Tothova Z, Levine R (2007). FLT3 mutations confer enhanced proliferation and survival properties to multipotent progenitors in a murine model of chronic myelomonocytic leukemia. Cancer Cell.

[75] Kelly L, Liu Q, Kutok J (2002). FLT3 internal tandem duplication mutations associated with human acute myeloid leukemias induce myeloproliferative disease in a murine bone marrow transplant model. Blood.

[76] Li L, Piloto O, Nguyen H (2008). Knock-in of an internal tandem duplication mutation into murine FLT3 confers myeloproliferative disease in a mouse model. Blood.

