# Supplementary material for: Calcium and Calcineurin-NFAT Signaling Regulate Granulocyte-Monocyte Progenitor Cell Cycle via Flt3-L
Source: Stem Cells. 2014 Nov 26;32(12):3232–44. doi: 10.1002/stem.1813 (PMC4282522; doi:10.1002/stem.1813)
Supplement: Supplementary file 1 — Supplementary [file stem0032-3232-SD1.pdf]

Supplemental Materials

Calcium and calcineurin-NFAT signaling regulate granulocyte-monocyte progenitor cell cycle via FLT3-L

Jan Fric, Clarice XF Lim, Alexandra Mertes, Bernett TK Lee, Elena Viganò, Jinmiao Chen, Francesca Zolezzi, Anis Larbi, Michael Poidinger, Herbert Strobl, Teresa Zelante and Paola Ricciardi-Castagnoli

Singapore Immunology Network (SIgN), Agency for Science, Technology and Research (A*STAR), Singapore

Supplemental Figures page 2

Supplemental Table page 11

Supplemental Methods page 13

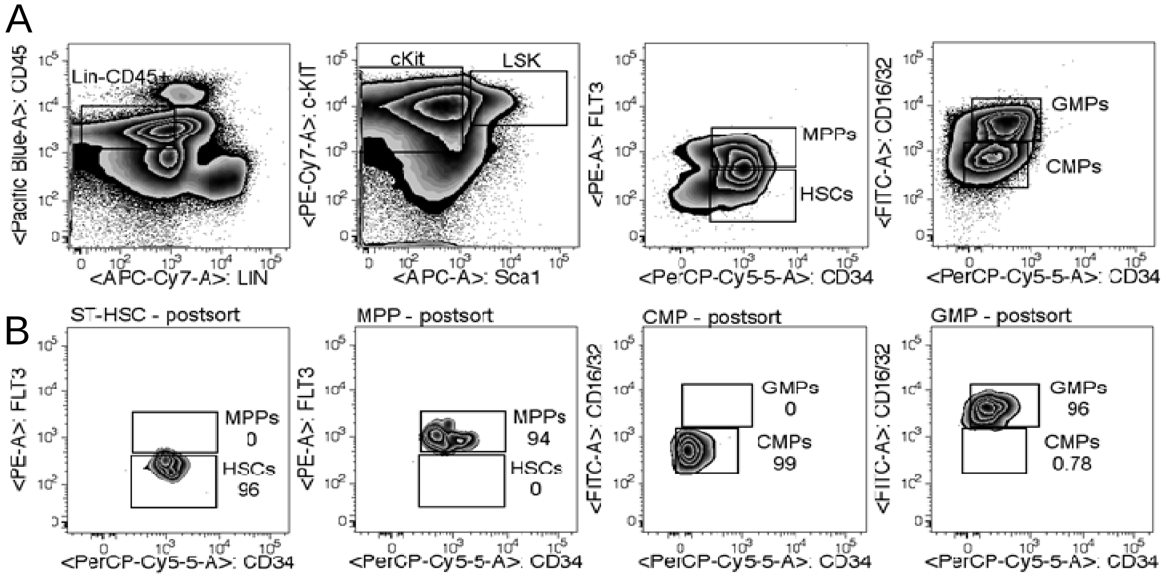

Supplemental Figure 1: Example of gating strategy (A) for analysis and sorting of haematopoietic progenitors and sorting purity (B). BM cells from host mice were harvested and labeled for identification of progenitors. Progenitor populations identified as LSKs (lin$^-$, cKit$^+$, Sca-1^{+-}), MPPs (lin$^-$, cKit$^+$, Sca-1$^+$, CD34$^+$, FLT3$^+$), CMPs (lin$^-$, cKit$^+$, Sca-1$^-$, CD34$^+$, CD16/32^{int}) and GMPs (lin$^-$, cKit$^+$, Sca-1$^-$, CD34$^+$, CD16/32^{high}). Sorting protocol was optimized and cells with purity exceeding 90% we used. In some experiments HSCs and MPPs were analyzed together, referred as LSKs.

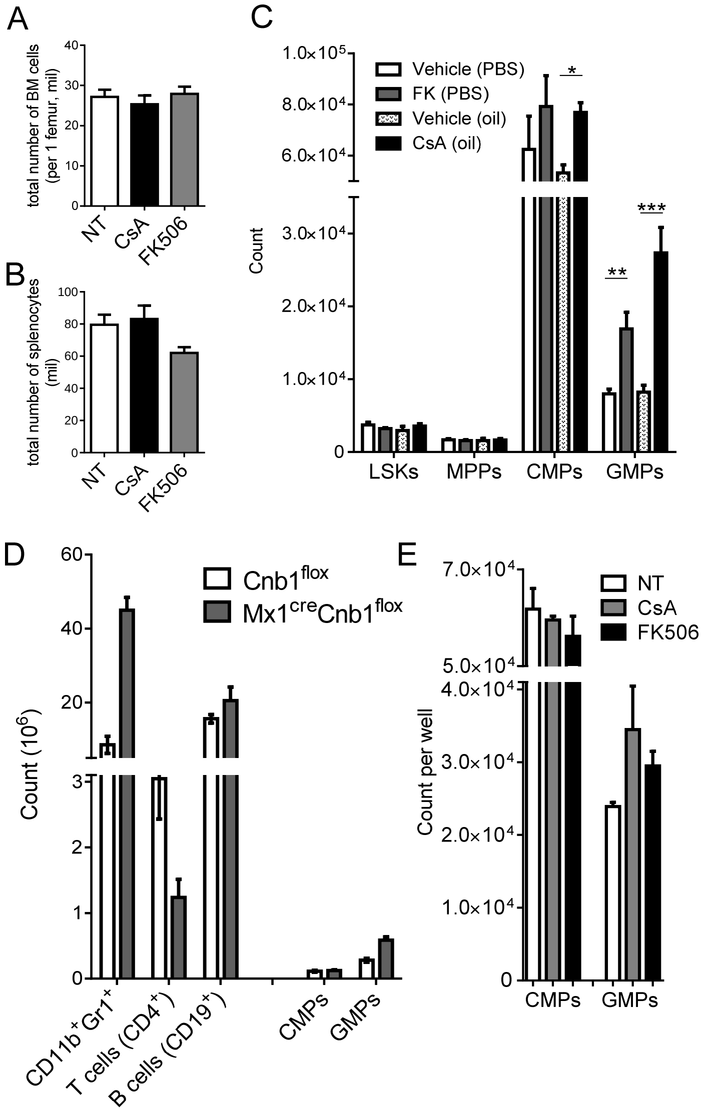

Supplemental Figure 2: Total numbers of progenitor cells in mice treated with calcineurin-NFAT inhibitors, in Cnb1$^{flox/flox}$Mx1-cre reconstituted mice and *in vitro* culture of sorted progenitors. Quantification of the total cell numbers in BM **(A)** and spleen **(B)** upon *in vivo* treatment with CsA and FK506. **(C)** Quantification of total numbers of LSKs, MPPs, CMPs and GMPs in BM upon calcineurin-NFAT inhibitors administration. **(D)** Quantification of total numbers of CD11b$^+$Gr1$^+$, T, B cells, CMPs and GMPs in mice reconstituted with BM from Cnb1$^{flox/flox}$Mx1-cre and CD45.1 control in ratio 1:1, cell counts were analyzed after full engraftment followed by KO induction with Poly I:C administration. **(E)** Total cell count in culture of sorted CMPs and GMPs treated with CsA and FK506. Values are shown as means ±SEM and unpaired Student's t-test was used to identify significant differences between groups (*denotes $p < 0.05$, **denotes $p < 0.01$ and ***denotes $p < 0.001$).

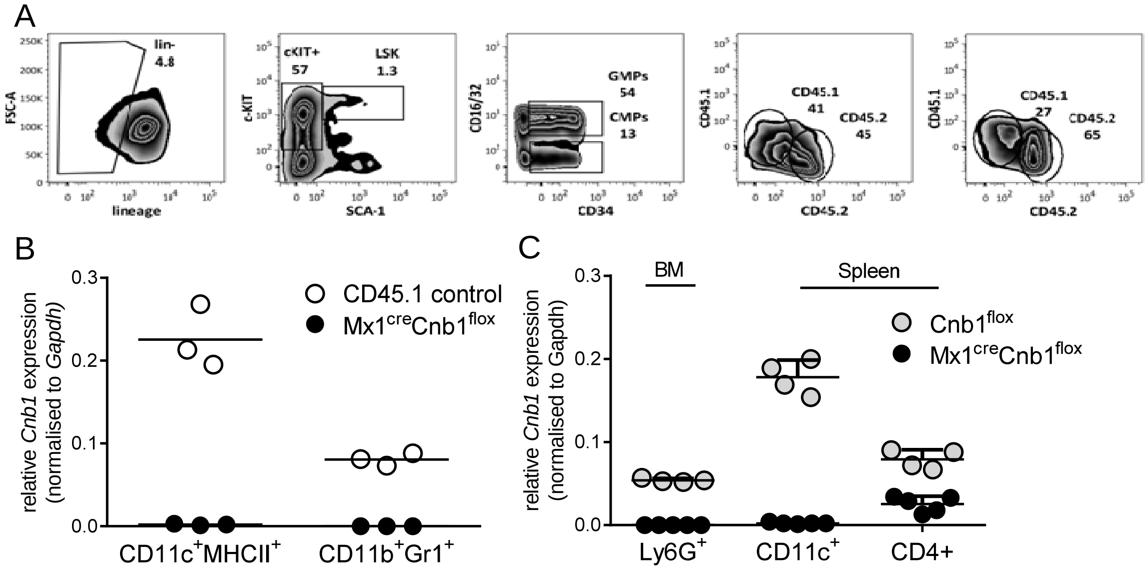

Supplemental Figure 3: (A) Example of gating strategy for analysis and sorting of haematopoietic progenitors from reconstituted chimeric mice. BM cells from host mice were harvested and labeled for identification of progenitors. Progenitor populations identified as LSKs (lin$^-$, cKit$^+$, Sca-1^{+-}), MPPs (lin$^-$, cKit$^+$, Sca-1$^+$, CD34$^+$, FLT3$^+$), CMPs (lin$^-$, cKit$^+$, Sca-1$^-$, CD34$^+$, CD16/32^{int}) and GMPs (lin$^-$, cKit$^+$, Sca-1$^-$, CD34$^+$, CD16/32^{high}), CD45.1 and CD45.2 markers were used in order to distinguish cells from each donor. **(B)** Cells from 3 mice were sorted to the indicated subsets and mRNA levels of Cnb1 were analyzed to assess the KO efficiency. **(C)** Conditional calcineurin KO mouse Cnb1$^{flox/flox}$Mx1-cre and littermate control Cnb1$^{flox/flox}$ Mx1-wt were analyzed for Cnb1 KO efficiency after Poly I:C injection. DCs (CD11c), neutrophils (Gr-1) and T (CD4) cells enriched with magnetic beads were analyzed by qPCR to confirm diminishing Cnb1 expression. Individual mice n=4 and mean from 1 of 3 representative experiments are plotted.

A Pathway Maps.

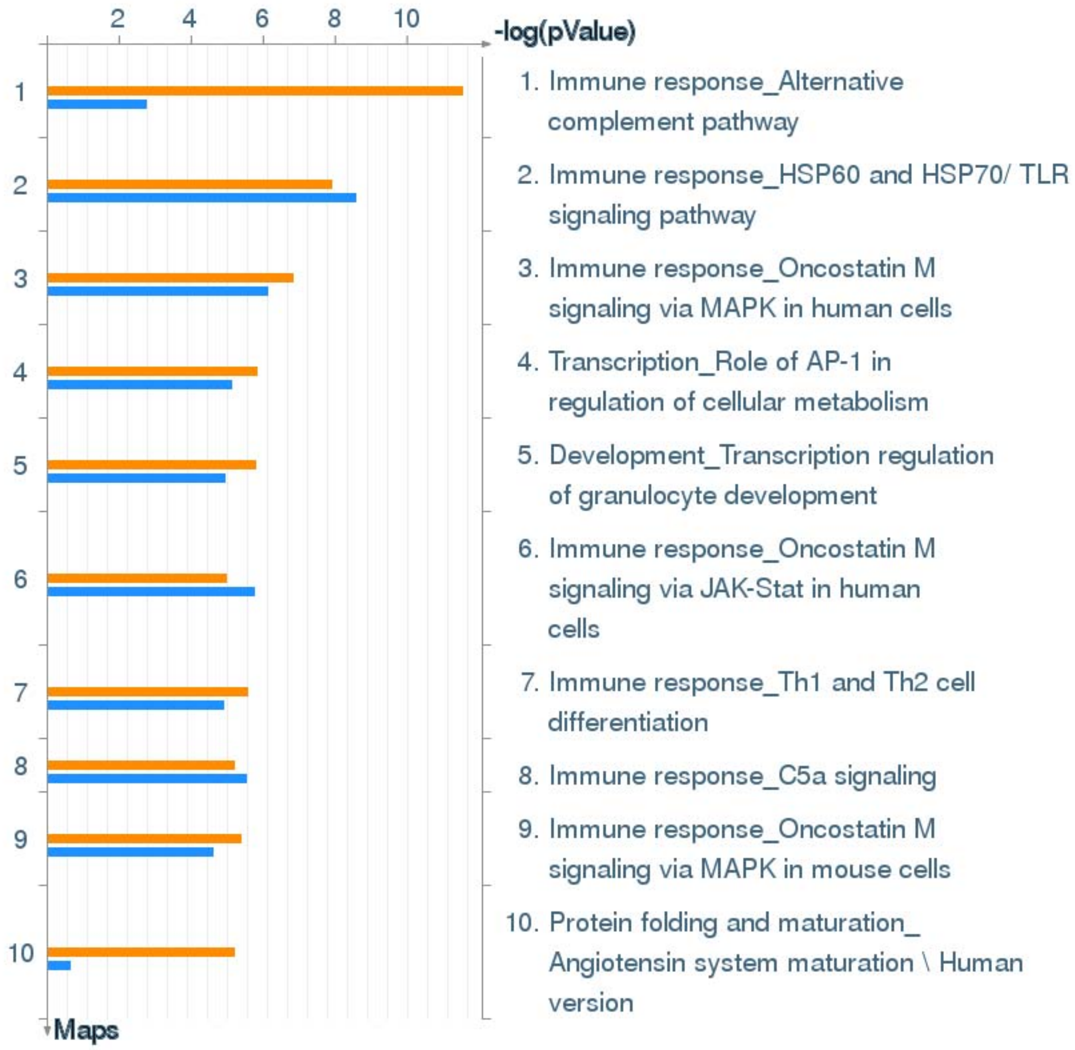

1.	(orange)	Control versus CsA treated cultured for 48h
2.	(blue)	Control versus CsA treated cultured for 24h

B GO Processes

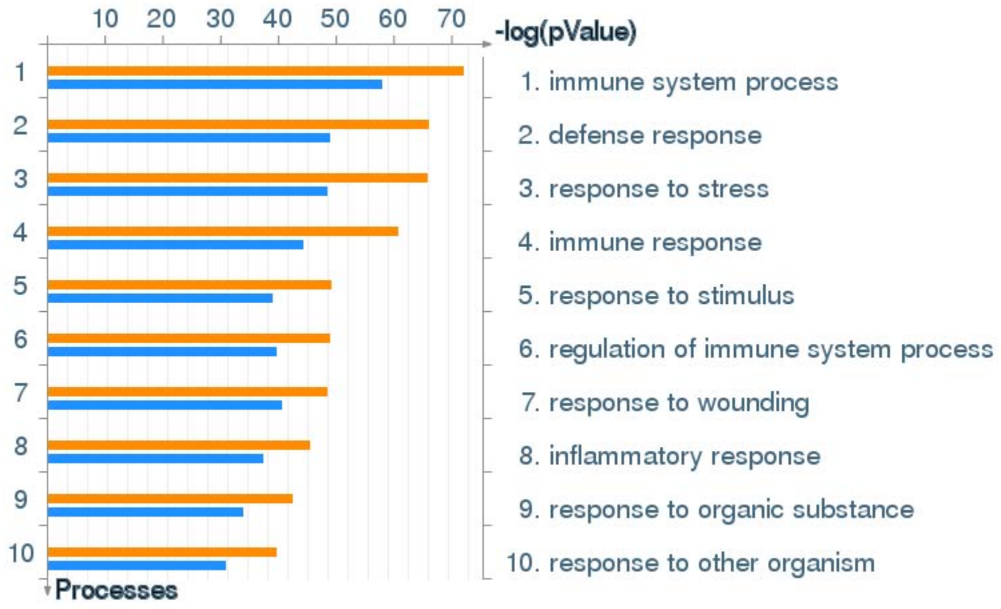

1.		Control versus CsA treated cultured for 48h
2.		Control versus CsA treated cultured for 24h

Supplemental Figure 4: Analysis of Pathways and Processes in cKIT-enriched progenitors cultured with calcineurin/NFAT inhibitors. The enrichment analysis was carried out by first mapping the differentially regulated genes to the “common”, “similar” and “unique” sets in MetaCore, allowing for the identification of MetaCore ontology terms which are subsequently enriched. The significance of the enrichment was estimated for the number of intersecting genes in the ontology term using the hypergeometric distribution. Two sets of ontologies representing canonical pathway maps (A) and cellular and molecular processes (B) were used in the analysis. These ontologies are the results of expert manual curation by Thomson Reuter scientists. The enrichment significance is shown as –log of the P-values in orange for the 48 hour time point and in blue for the 24 hour time point for both ontologies in the figure below. Significant ontology terms are ranked by their P-values.

A Molecular and cellular Functions

Name	p-value	# Molecules
Cell Cycle	8.65E-08 - 1.20E-02	118
Cellular Growth and Proliferation	3.19E-07 - 1.15E-02	238
Gene Expression	5.69E-07 - 5.65E-03	166
Cellular Development	6.34E-07 - 1.13E-02	196
Cell Death and Survival	6.91E-07 - 1.14E-02	232

B Physiological System Development and Function

Physiological System Development and Function

Name	p-value	# Molecules
Embryonic Development	1.47E-05 - 1.16E-02	69
Connective Tissue Development and Function	1.88E-05 - 1.13E-02	57
Tissue Development	5.60E-05 - 1.16E-02	95
Hematological System Development and Function	6.78E-05 - 1.11E-02	105
Hematopoiesis	7.17E-05 - 1.11E-02	77

C Networks

Top Networks

ID	Associated Network Functions	Score
1	Cell Signaling, Cell Death and Survival, Tissue Morphology	48
2	Cancer, Skeletal and Muscular Disorders, Tissue Morphology	48
3	Cellular Assembly and Organization, Post-Translational Modification, Developmental Disorder	43
4	Cancer, Dermatological Diseases and Conditions, Hereditary Disorder	38
5	Hereditary Disorder, Neurological Disease, Cardiac Enlargement	34

Supplemental Figure 5: Analysis of Pathways and Processes of PBMCs of control and CsA treated patients. The raw microarray data in the form of CEL files for GEO series GSE22224 (http://www.ncbi.nlm.nih.gov/geo/query/acc.cgi?acc=GSE22224) was down-loaded from NCBI and processed using R and Bioconductor. The affy package was used to process the CEL files and the expression data normalized using RMA. The normalized log2 transformed expression data was then analyzed in the Limma package comparing CsA treatment and healthy controls. This analysis revealed a total of 870 genes which are differentially expressed in CsA treated patients compared to healthy controls. These 870 differentially expressed genes were then used in a standard IPA analysis and the enrichment results for "Molecular and Cellular Functions", "Physiological System Development and Function" and "Networks" shown in the figure above.

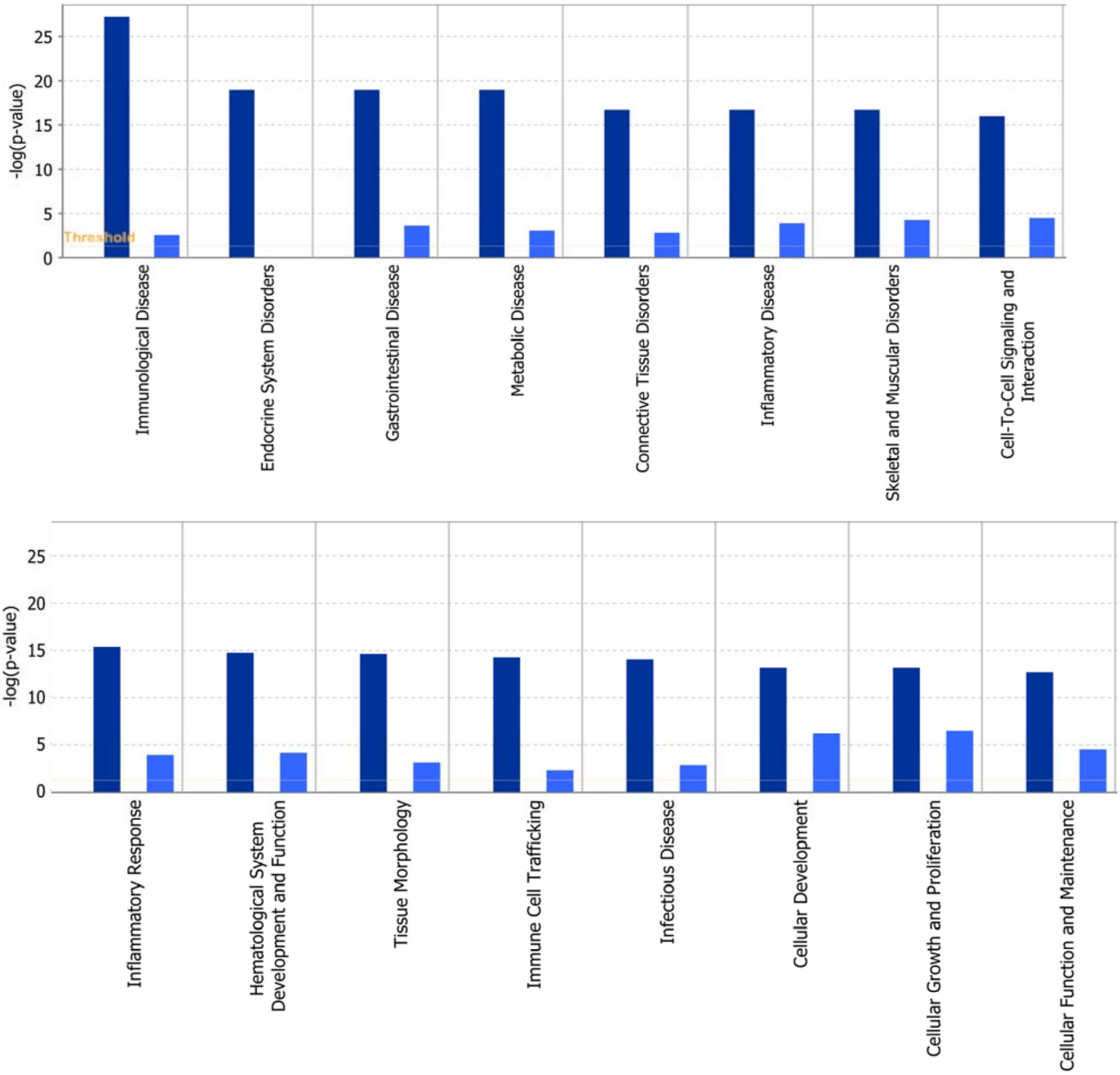

Supplemental Figure 6: IPA comparison processes induced in patients PBMCs (light blue) and in mouse myeloid progenitors (dark blue) upon CsA treatment. The IPA analysis results for the 870 differentially expressed genes in GSE22224 described in Supplemental Figure 5 was compared to a similar IPA analysis done for the differentially expressed genes for treatment in Supplemental Table 1. A number processes were found to be similar in both IPA analysis as shown in the figure above.

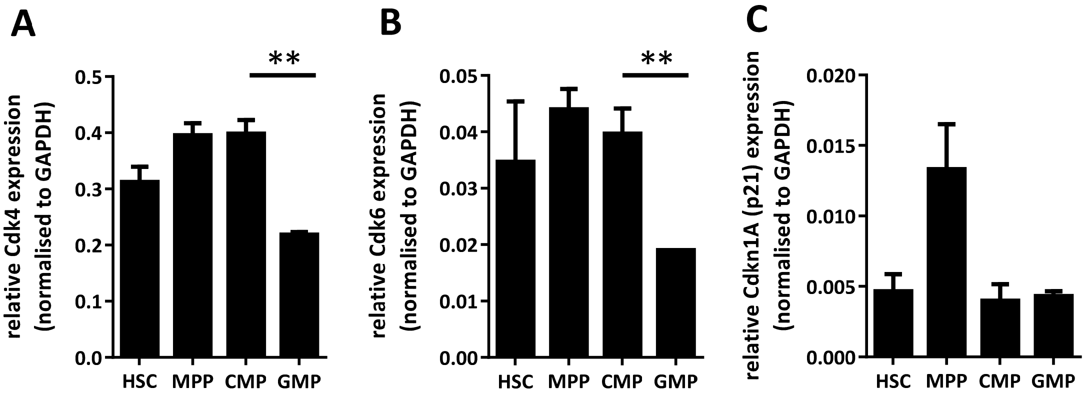

Supplemental Figure 7: Granulocyte monocyte progenitors down regulate expression of cell cycle kinases *Cdk4* and *Cdk6*. Gene expression of *Cdk4* **(A)**, *Cdk6* **(B)**, *Cdkn1a* (*p21*) **(C)** in HSCs, MPPs, CMPs and GMPs was measured by qPCR. mRNA was isolated immediately after sorting without any cell culture. Data are presented as mean ±SE from at least 3 independent experiments, in which BM cells from at least 15 mice were pooled for sorting. Values are shown as means ±SEM and unpaired Student's t-test was used to identify significant differences between groups (**denotes $p < 0.01$).

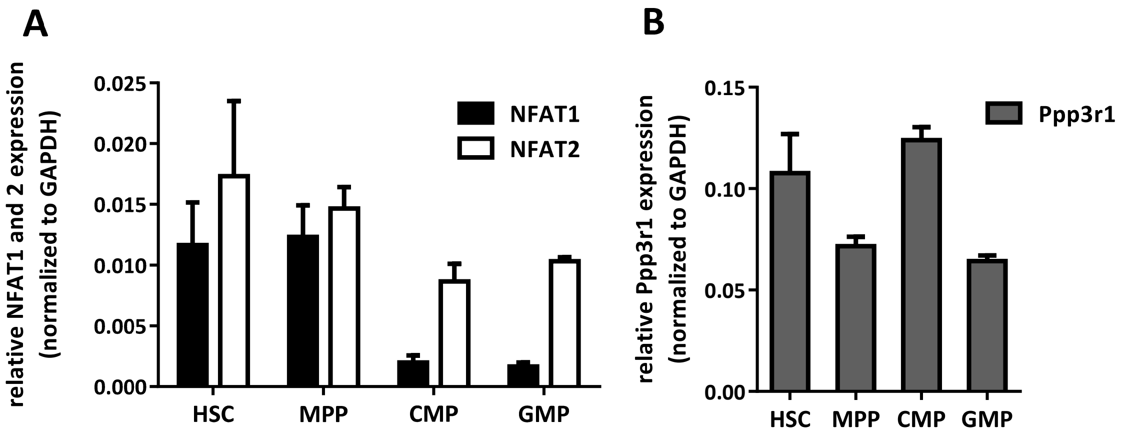

Supplemental Figure 8: *Nfat1, Nfat2* and calcineurin (*Ppp3r1*) are expressed in sorted progenitors. Gene expression of *Nfat1,2* **(A)** and regulatory calcineurin subunit B (*Cnb1, Ppp3r1*) **(B)** in HSCs, MPPs, CMPs and GMPs was measured by qPCR. mRNA was isolated immediately after sorting without any cell culture. Data are presented as mean ±SE from at least 3 independent experiments, in which BM cells from at least 15 mice were pooled for sorting.

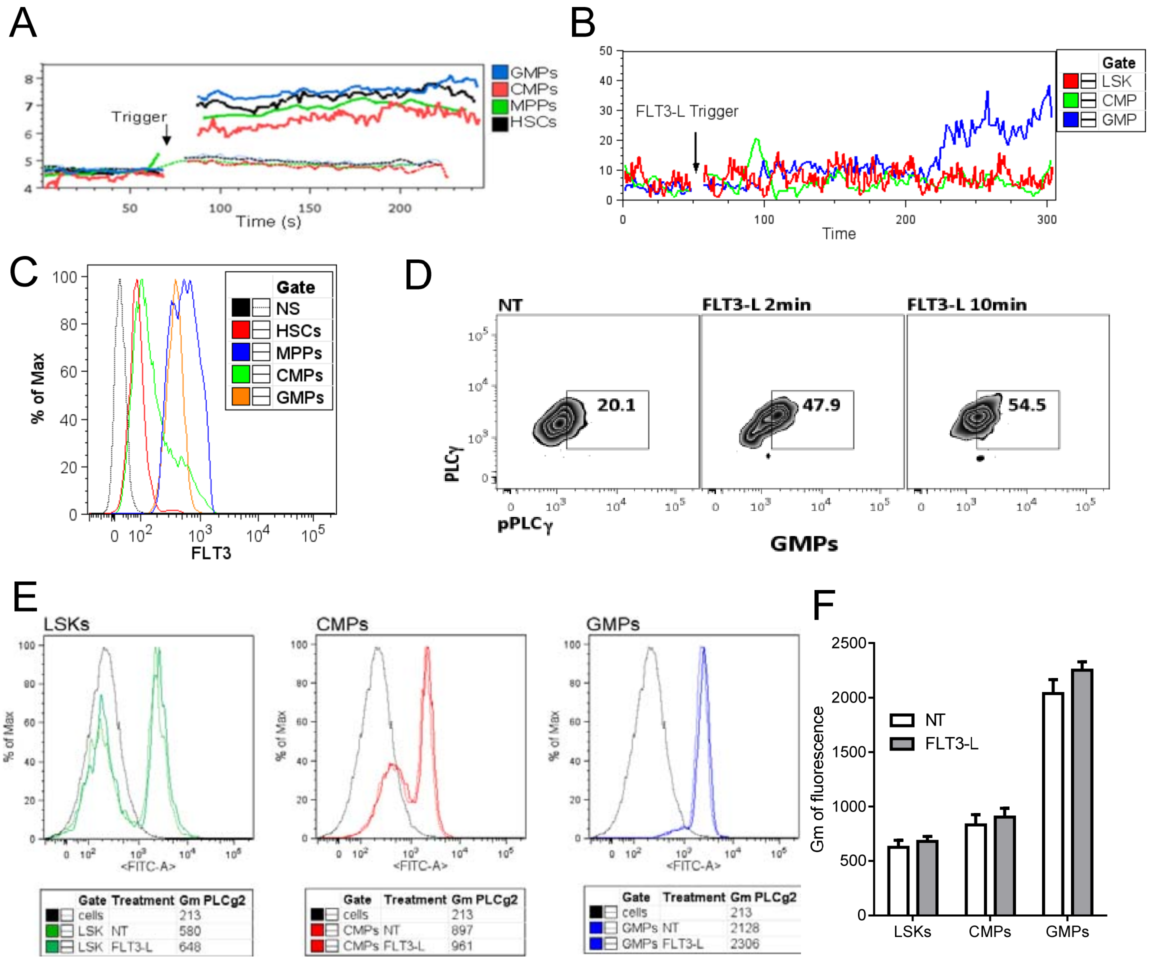

Supplemental Figure 9: Myeloid progenitors respond to ionomycin and FLT3-L with different sensitivity. (A) Representative graph of intracellular Ca^{2+} release analysis in lineage-depleted BM cells with progenitor subpopulations identified as HSCs, MPPs, CMPs and GMPs. INDO-1 loaded progenitors are compared for changes in intracellular Ca^{2+} after trigger with **(A)** ionomycin (500 ng/ml) or **(B)** FLT3-L (2µg/ml) added after 1 min of measurement. Data are taken from 1 of 4 independent experiments, in which BM cells from at least 5 mice were pooled. **(C)** Expression of FLT3 on the surface of different progenitors. **(D)** Phosporylation of $PLC\gamma_1$ in GMPs upon trigger with FLT3-L, showing phosphorylated portion of $PLC\gamma_1$ ($pPLC\gamma_1$-FITC) versus total $PLC\gamma_1$-PE staining. **(E)** Changes in phorsporylation of $PLC\gamma_2$ ($pPLC\gamma_2$-FITC), dotted black line – unstained, dotted colored line – untreated and full colored line triggered with FLT3-L, each histogram for different progenitors type (LSKs - green, CMPs - red, GMPs - blue). **(F)** Gm for $pPLC\gamma_2$-FITC after FLT3-L trigger.

Supplemental Table 1

	Timepoint		treatment		timepoint:treatment	
**symbol**	**P-value**	**P-value (adj)**	**P-value**	**P-value (adj)**	**P-value**	**P-value (adj)**
Il4ra	2.59E-02	3.91E-02	1.38E-06	1.21E-04	1.27E-02	6.23E-02
Il1rn	6.67E-06	1.35E-04	5.47E-06	2.41E-04	4.28E-03	4.18E-02
Il1r2	3.06E-07	2.69E-05	9.09E-06	2.67E-04	1.40E-02	6.32E-02
Cdkn2d	2.38E-03	5.65E-03	2.05E-05	4.51E-04	4.04E-01	5.46E-01
Rara	9.50E-04	2.99E-03	3.84E-05	6.76E-04	1.31E-01	2.33E-01
Il6st	3.87E-01	4.10E-01	5.95E-05	7.94E-04	9.13E-01	9.23E-01
Ccnd3	2.54E-01	2.85E-01	7.06E-05	7.94E-04	8.84E-01	9.04E-01
Itga1	3.62E-01	3.88E-01	7.21E-05	7.94E-04	8.67E-01	8.98E-01
Il13ra1	3.21E-04	1.28E-03	1.21E-04	1.08E-03	4.84E-01	6.00E-01
Cx3cr1	4.07E-03	8.52E-03	1.22E-04	1.08E-03	8.24E-02	1.67E-01
Irak3	5.85E-04	2.15E-03	1.51E-04	1.21E-03	2.71E-02	8.28E-02
Cdk5rap3	1.83E-01	2.15E-01	1.72E-04	1.26E-03	6.19E-02	1.40E-01
Irf1	3.93E-05	2.58E-04	2.03E-04	1.30E-03	7.49E-04	3.31E-02
Cd14	1.86E-04	9.62E-04	2.07E-04	1.30E-03	7.94E-01	8.74E-01
Fcgr2b	2.38E-05	2.58E-04	2.86E-04	1.68E-03	1.52E-03	3.31E-02
Stat1	4.23E-05	2.58E-04	3.08E-04	1.70E-03	8.37E-03	5.26E-02
Hexim2	2.75E-05	2.58E-04	4.55E-04	2.36E-03	5.63E-01	6.58E-01
Cdk1	1.90E-03	4.77E-03	5.09E-04	2.49E-03	6.73E-03	4.94E-02
Cnnm4	8.37E-03	1.47E-02	6.12E-04	2.83E-03	2.16E-03	3.31E-02
Il18	1.88E-03	4.77E-03	6.51E-04	2.86E-03	2.81E-01	4.24E-01
H2-Eb1	9.23E-06	1.35E-04	7.91E-04	3.17E-03	4.79E-02	1.21E-01
Cxcr2	1.15E-02	1.99E-02	8.26E-04	3.17E-03	5.68E-01	6.58E-01
Ccna2	3.54E-05	2.58E-04	8.29E-04	3.17E-03	2.92E-03	3.67E-02
Ets1	1.44E-03	3.92E-03	8.75E-04	3.21E-03	1.99E-01	3.13E-01
Il1b	1.09E-03	3.20E-03	1.02E-03	3.59E-03	2.82E-02	8.28E-02
Nfil3	1.09E-01	1.35E-01	1.75E-03	5.71E-03	5.64E-02	1.34E-01
Notch1	2.53E-01	2.85E-01	2.93E-03	9.20E-03	1.26E-02	6.23E-02
Nfkb2	2.59E-01	2.85E-01	3.20E-03	9.71E-03	9.59E-02	1.84E-01
E2f7	4.50E-02	6.09E-02	3.61E-03	1.06E-02	8.59E-01	8.98E-01
Ccnf	5.54E-03	1.13E-02	4.36E-03	1.20E-02	4.80E-02	1.21E-01
Cnnm2	9.90E-02	1.25E-01	4.37E-03	1.20E-02	3.58E-01	5.08E-01
Elf4	8.36E-01	8.36E-01	4.65E-03	1.24E-02	3.48E-01	5.02E-01
Cd209a	6.10E-01	6.32E-01	4.96E-03	1.28E-02	2.84E-01	4.24E-01
Il6ra	7.95E-01	8.04E-01	5.23E-03	1.31E-02	1.98E-01	3.13E-01
Hpgds	2.99E-03	6.93E-03	5.66E-03	1.38E-02	8.09E-01	8.79E-01
Itgam	8.45E-06	1.35E-04	6.02E-03	1.43E-02	1.32E-01	2.33E-01
Stat3	9.07E-02	1.16E-01	8.26E-03	1.91E-02	7.60E-01	8.57E-01
Cd34	1.47E-03	3.92E-03	8.72E-03	1.93E-02	4.43E-01	5.82E-01
Il15	6.01E-03	1.17E-02	8.78E-03	1.93E-02	7.78E-01	8.66E-01
Fas	2.18E-01	2.53E-01	1.24E-02	2.65E-02	5.14E-01	6.28E-01
Nfatc2	4.40E-05	2.58E-04	1.30E-02	2.73E-02	4.66E-01	5.98E-01

Stat6	6.87E-03	1.26E-02	1.36E-02	2.79E-02	9.48E-01	9.48E-01
Il10ra	2.75E-04	1.21E-03	1.49E-02	2.91E-02	1.35E-01	2.33E-01
Irf2	5.79E-03	1.16E-02	1.93E-02	3.63E-02	5.43E-01	6.49E-01
Ccno	1.40E-02	2.28E-02	1.95E-02	3.63E-02	5.45E-01	6.49E-01
E2f8	3.76E-02	5.25E-02	1.98E-02	3.63E-02	7.27E-02	1.56E-01
**Cdk4**	**1.87E-05**	**2.35E-04**	**2.07E-02**	**3.72E-02**	**1.29E-03**	**3.31E-02**
Etv1	4.39E-02	6.03E-02	2.13E-02	3.73E-02	3.20E-01	4.69E-01
Cd40	3.97E-03	8.52E-03	2.16E-02	3.73E-02	1.24E-02	6.23E-02
Fcgr1	2.51E-06	7.36E-05	2.23E-02	3.78E-02	1.23E-01	2.26E-01
Irf5	1.28E-02	2.16E-02	2.31E-02	3.83E-02	1.39E-01	2.36E-01
Ccnb1	8.03E-03	1.35E-02	2.81E-02	4.34E-02	3.30E-02	9.34E-02
Ccne1	3.72E-02	5.25E-02	3.28E-02	5.34E-02	8.10E-02	1.67E-01
Csf3r	1.54E-02	2.42E-02	3.59E-02	5.64E-02	1.07E-01	1.99E-01
Cebpe	6.14E-03	1.17E-02	3.71E-02	5.73E-02	3.87E-01	5.37E-01
Cdk18	1.40E-06	6.18E-05	3.87E-02	5.87E-02	5.77E-01	6.59E-01
Hmox2	6.04E-01	6.32E-01	4.14E-02	6.17E-02	4.81E-01	6.00E-01
Ccny	1.27E-01	1.55E-01	4.28E-02	6.27E-02	9.03E-02	1.77E-01
Nfia	2.91E-02	4.20E-02	6.05E-02	8.57E-02	8.64E-01	8.98E-01
**Cdk6**	**8.36E-03**	**1.47E-02**	**6.14E-02**	**8.57E-02**	**6.46E-02**	**1.42E-01**
Stat2	3.82E-05	2.58E-04	6.27E-02	8.63E-02	1.87E-02	7.13E-02
Csf1r	2.96E-04	1.24E-03	6.47E-02	8.75E-02	2.95E-02	8.38E-02
Cebpb	7.22E-01	7.39E-01	7.17E-02	9.56E-02	4.69E-01	5.98E-01
Cdc23	6.62E-04	2.24E-03	7.33E-02	9.62E-02	7.94E-03	5.26E-02
Il2ra	1.48E-01	1.76E-01	8.45E-02	1.09E-01	2.44E-02	7.96E-02
Irf7	6.48E-04	2.24E-03	1.01E-01	1.29E-01	3.91E-01	5.37E-01
Ppp1r3b	2.71E-02	3.98E-02	1.10E-01	1.39E-01	1.76E-01	2.92E-01
E2f6	2.14E-04	1.05E-03	1.17E-01	1.45E-01	8.35E-02	1.67E-01
E2f2	1.37E-01	1.65E-01	1.24E-01	1.52E-01	8.68E-01	8.98E-01
Kit	8.99E-02	1.16E-01	1.32E-01	1.59E-01	4.14E-01	5.53E-01
Cdk2ap1	1.17E-03	2.96E-03	1.71E-01	2.01E-01	1.15E-01	1.97E-01
Irf9	2.71E-04	1.21E-03	1.76E-01	2.10E-01	3.74E-02	9.98E-02
Cd59b	1.48E-02	2.37E-02	1.84E-01	2.16E-01	2.75E-02	8.28E-02
Egr1	2.57E-01	2.85E-01	3.19E-01	3.70E-01	2.18E-02	7.69E-02
Cdc16	5.83E-04	2.15E-03	4.38E-01	4.95E-01	1.05E-03	3.31E-02
Ikzf1	6.60E-02	8.66E-02	4.60E-01	5.13E-01	1.61E-02	6.76E-02
Irf4	2.62E-02	3.91E-02	4.67E-01	5.14E-01	1.79E-02	7.13E-02
Cks2	6.48E-03	1.21E-02	5.27E-01	5.72E-01	1.98E-02	7.27E-02
Ccnh	1.32E-02	2.19E-02	7.21E-01	7.73E-01	5.06E-02	1.24E-01
Pa2g4	3.94E-05	2.58E-04	7.35E-01	7.80E-01	6.65E-03	4.94E-02
Cd24a	3.20E-01	3.48E-01	7.62E-01	7.98E-01	1.44E-02	6.32E-02
Hcst	1.02E-03	3.10E-03	9.17E-01	9.49E-01	1.81E-01	2.95E-01
E2f3	3.12E-03	7.04E-03	9.36E-01	9.53E-01	4.20E-03	4.18E-02
Nfatc3	1.21E-03	3.42E-03	9.42E-01	9.53E-01	2.44E-02	7.96E-02
Il3ra	5.12E-02	6.83E-02	9.94E-01	9.94E-01	2.26E-03	3.31E-02

Supplemental Table 1: Microarray gene expression data Table showing the results of the 2 way ANOVA for the microarray gene expression data. The factors of time and treatment together with the interaction term were tested in the 2 way ANOVA, with multiple testing corrections carried out using the method of Benjamini and Hochberg. Nominal and multiple testing corrected P values are presented in the table as “P-value” and “P-value (adj)” respectively.

Supplemental Materials and Methods:

Microarray hybridization and analysis. Total RNA was extracted using a double extraction protocol: RNA isolation by acid guanidinium thiocyanate-phenol-chloroform extraction (Trizol Invitrogen) followed by a Qiagen RNeasy clean-up procedure. Total RNA integrity was measured by Agilent Bioanalyzer and the RNA Integrity Number (RIN) was calculated; samples with RIN greater than 9 were used for microarray analysis. ssDNA was prepared, fragmented and labelled according to the Affymetrix protocol. Fragmented ssDNAs were hybridized to the standard arrays for 17h at 45°C; the arrays were then washed and stained using the fluidics station and then scanned using GeneChip Scanner 3000. Images were analyzed using Command Console and comparison analyses were carried out according to the instructions provided by Affymetrix. Gene expression data generated on Affymetrix GeneChip Mouse Gene ST 1.0 TS arrays were processed using Bioconductor version 2.11 running on R version 2.15.2. Normalization of the arrays employed RMA and the resulting log2 transformed values were used for all subsequent analysis. The gene expression data were then filtered for only probes where the associated gene had a valid NCBI Entrez Gene ID to restrict data to well annotated genes. Gene ontology terms were used to identify genes involved in regulation of cell cycle and transcriptional regulation of differentiation and hematopoiesis. These genes were then tested using a series of 2 way ANOVAs to identify genes that differed in their expression levels due to time or treatment. Processing of the data used Accelrys Pipeline Pilot with visualizations in TIBCO Spotfire. All microarray data files are available for free download at the Gene Expression Omnibus (GEO accession number: GSE47208, "http://www.ncbi.nlm.nih.gov/geo".

Quantitative real-time PCR primers

Gapdh: Forward 5’-TCG TCC CGT AGA CAA AAT GG-3’; Reverse 5’-TTG AGG TCA ATG AAG GGG TC-3’, *Nfat1*: Forward 5’-CTG GTC TAC GGG GGC CAG CA-3’, Reverse 5’-GGC AGG GAC TGG GTG GTA GG-3’, *Nfat2*: Forward 5’-TGC AAG CCA AAT TCC CTG GTG G-3’ Reverse 5’-GGG GTC GGG AGG CAT GGT GA-3’, *Nfat3*: Forward 5’-ATG GTG GCT ACA GCC AGC TAT GAA-3’ Reverse’-TGG CAG GAA GTT GGA ACC AGT CAA-3’ *Nfat4*: Forward 5’-ACC TCA TTG GGA GGC TGA AGG AAA-3’ Reverse 5’-TAT GCT GGC TGC ACT TGA CAA AGC-3’ *Cdk4*: Forward 5’-TTG TGC AGG TAG GAG TGC TG-3’ Reverse 5’-TGC CAG AGA TGG AGG AGT CT-3’, *Cdkn1a (p21)*: Forward 5’-ATC ACC AGG ATT GGA CAT GG-3’ Reverse 5’-CGG TGT CAG AGT CTA GGG GA-3’, *Cdk6*: Forward 5’-GCT TGG TTT CTC TGT CCG TC-3’, Reverse 5’-GTG CTG AGG CAC CTG GAG-3’, *Cnb1*: Forward 5’-TG TTC CGT GCC TTG AGG TTG-3’; Reverse 5’-TCT GTT CCT TAT CGC CTT TGA C-3’
